# Aza-BODIPY based carbonic anhydrase IX: Strategy to overcome hypoxia limitation in photodynamic therapy

**DOI:** 10.3389/fchem.2022.1015883

**Published:** 2022-11-02

**Authors:** Thitima Pewklang, Kantapat Chansaenpak, Siti Nursyahirah Bakar, Rung-Yi Lai, Chin Siang Kue, Anyanee Kamkaew

**Affiliations:** ^1^ School of Chemistry, Institute of Science, Suranaree University of Technology, Nakhon Ratchasima, Thailand; ^2^ National Nanotechnology Center, National Science and Technology Development Agency, Thailand Science Park, Pathum Thani, Thailand; ^3^ Faculty of Health and Life Sciences, Management and Science University, Shah Alam, Selangor, Malaysia

**Keywords:** aza-BODIPY, PDT, acetazolamide, carbonic anhydrase IX (CA9), hypoxia

## Abstract

Hypoxia caused by photodynamic therapy (PDT) is a major hurdle to cancer treatment since it can promote recurrence and progression by activating angiogenic factors, lowering therapeutic efficacy dramatically. In this work, AZB-I-CAIX_2_ was developed as a carbonic anhydrase IX (CAIX)-targeting NIR photosensitizer that can overcome the challenge by utilizing a combination of CAIX knockdown and PDT. AZB-I-CAIX_2_ showed a specific affinity to CAIX-expressed cancer cells and enhanced photocytotoxicity compared to AZB-I-control (the molecule without acetazolamide). Moreover, selective detection and effective cell cytotoxicity of AZB-I-CAIX_2_ by PDT in hypoxic CAIX-expressed murine cancer cells were achieved. Essentially, AZB-I-CAIX_2_ could minimize tumor size in the tumor-bearing mice compared to that in the control groups. The results suggested that AZB-I-CAIX_2_ can improve therapeutic efficiency by preventing PDT-induced hypoxia through CAIX inhibition.

## 1 Introduction

Photodynamic therapy (PDT) is becoming increasingly popular as a non-invasive cancer treatment in clinical settings (Lucky et al., 2015; Jung et al., 2017a). In PDT, a photosensitizer (PS) can be activated by a specific wavelength of light to generate reactive oxygen species (ROS), including singlet oxygen (^1^O_2_) to harm the cells directly through apoptosis and indirectly *via* tumor vasculature shutdown induction and immune mediators recruitment (Henderson and Dougherty, 1992; Dougherty et al., 1998; DeRosa and Crutchley, 2002). Generally, PDT provides more advantages over other conventional cancer treatment approaches including localization improvement, minimally invasive technique, and repeatable without significant side effects (Dolmans et al., 2003; Jung et al., 2017a). Therefore, PDT provides a safe and effective treatment to selectively clear targeted cells or tissues such as cancer cells while avoiding toxic and side effects on normal or healthy tissues (Hong et al., 2016). However, most PDT agents predominantly function through the type II pathway (energy transfer), a highly oxygen-dependent mechanism that can lead to severe tumor hypoxia when the inner region of a tumor has < 20 mmHg O_2_ pressure due to insufficient blood supply (Ding et al., 2011; Krzykawska-Serda et al., 2014; Song et al., 2016). PDT-induced hypoxia can restrict the therapeutic effects of PDT and reduce its efficacy in tumor eradication because it triggers the hypoxia-inducing factor (HIF) mediated signaling cascade that upregulates several regulator genes to promote tumor growth (Freitas and Baronzio, 1991; Koukourakis et al., 2001; Huang, 2005). Therefore, developing a PDT system to avoid PDT-induced hypoxia is crucial for improving treatments.

Carbonic anhydrase (CA) has recently been recognized as a valuable target for the development of a variety of diagnostic and therapeutic agents for cancer, due to its overexpression in cancer and as a marker for tumor survival (Supuran, 2008; Mahalingam et al., 2018). Lately, it has been shown that two cell surface CA isoforms namely CA IX (almost exclusively associated with tumors) and CA XII (overexpressed in some tumor types) are overexpressed in many tumors and prominently associated with tumor progression (Supuran and Winum, 2015a). Since many CA isoforms (including CA IX) are linked to anion exchangers or sodium bicarbonate cotransporters, they play an important role in ion transport and electrolyte secretion in a variety of tissues and organs (Benej et al., 2014). The transcription factor HIF-1 binds to a gene’s hypoxia-responsive region to regulate the expression of multiple genes, including those producing CA IX. The wild-type von Hippel-Lindau tumor suppressor protein (pVHL) inhibits this process (Benej et al., 2014; McDonald and Dedhar, 2014). Hypoxia, *via* the HIF-1 cascade, causes high overexpression of CA IX in many tumors resulting in a pH drop in the tissue. Most hypoxic tumors are acidic (pH = 6), while normal tissues are neutral (pH = 7.4). Tumor cells decrease their pH both by the production of lactic acid (owing to the high glycolysis rate) and by CO_2_ hydration catalyzed by the tumor-associated CA IX isoform, which possesses an extracellular catalytic domain (Helmlinger et al., 2002). Low pH has been associated with tumorigenic transformation, chromosomal rearrangements, extracellular matrix breakdown, migration and invasion, induction of cell growth factors, and protease activation (Thiry et al., 2006). There are some small molecules that could inhibit the catalytic activity of CA. Two major classes of inhibitors that bind to the active site can be distinguished: the inhibitors which coordinate the zinc ion such as sulfonamide, sulfamate, sulfamide, dithiocarbamates, xanthate, and inorganic anions and the compounds which do not interact with the metal ion such as phenols, polyamines, and coumarins (Supuran and Winum, 2015a). Many potent CA inhibitors derived from acetazolamide, ethoxzolamide, and benzenesulfonamides have been reported to inhibit the growth of several tumor cells *in vitro* and *in vivo* (De Simone et al., 2006; Winum et al., 2008; Supuran, 2010; Supuran and Winum, 2015b; Wichert and Krall, 2015). CAIX-selective sulfonamide inhibitors reduced acidification of the medium, by inhibiting the catalytic activity of the enzyme and thus the generation of H^+^ ions, and bound only to hypoxic cells expressing CAIX. Under hypoxic conditions, tumor cells switch on proangiogenic factors such as EGFR and VEGF to promote angiogenesis for regrowth (Krock et al., 2011). Therefore, inhibition of CAIX enzymatic activity with targeted PDT conjugate could alleviate hypoxic mediated tumor growth and angiogenesis (Pucelik et al., 2020; Liang et al., 2021; Wan et al., 2021; Zhao et al., 2021; Zhou et al., 2022).

To mitigate oxygen depletion during PDT, CAIX inhibitors have recently been offered in tandem with PDT. For example, pyropheophorbide-a derivatives containing sulfonamide moiety showed favourable photo-cytotoxic effects *in vitro* and *in vivo* towards CAIX overexpression (Wang et al., 2022). In another study, the first rhenium (I)-based pyroptosis inducer, anchoring CAIX, was introduced for PDT in hypoxia and immunogenic tumor environments (Su et al., 2022). This offers a very promising tool for photodynamic immunotherapy. Due to the toxicity and depth of tissue penetration of the activating light, the utilization of metal complexes or NIR photosensitizers with low extinction coefficients is the main subject of concern in PDT.

**FIGURE 1:**
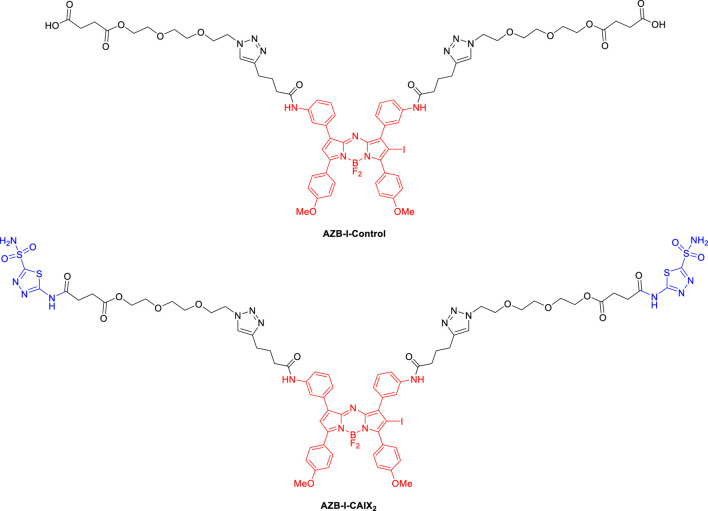
Structures of targeting probe (AZB-I-CAIX_2_) and control (AZB-I-control) in this study.

In this study, we used an aza-BODIPY photosensitizer in combination with CA pan-inhibitor, acetazolamide (AZ), to reprogram the hypoxic metabolism that aimed to overcome oxygen deficiency in PDT. Figure 1 shows the structures of the targeting probe (AZB-I-CAIX_2_) and the control (AZB-I-control) in this work. Aza-BODIPY dye could be an ideal PDT agent because it can absorb light in the near IR region since PDT can be more effective if near-IR absorbing photosensitizers (λmax > 700 nm) are used to treat deep-seated tissue (Grosjean et al., 1998). Moreover, their structures are synthetic accessible to conjugate with many targeting ligands (Kamkaew and Burgess, 2013; Kamkaew et al., 2013; Kamkaew and Burgess, 2015; Kue et al., 2015). Because the bivalent ligand was shown to have superior selectivity to CAIX (Krall et al., 2014), we attached two AZ moieties to the photosensitizer for our targeting probe. Furthermore, when compared to parent acetazolamide, increasing the tail of AZ resulted in improved Ki over hCA IX (Turkmen et al., 2005; Waghorn et al., 2012; Teruya et al., 2016). Moreover, the linker was used in the conjugation to improve the compound’s binding affinity and hydrophilicity.

## 2 Experimental section

### 2.1 Chemistry experimental instruments and synthesis

For all reactions, glassware was oven-dried before use. All the reagents werepurchased from commercial sources and used without further purification. ^1^H, ^13^C, and ^19^F NMR spectra were recorded on a Bruker-500 MHz spectrometer at room temperature. Chemical shifts (δ, ppm) of ^1^H NMR spectra were reported in CDCl_3_, MeOD-d_4_, and DMSO-d_6_ and coupling constant (*J*, Hz). Mass spectra (MS) were measured under high-resolution ESI conditions. All the UV/vis absorption spectra and fluorescence were recorded on a UV-vis Spectrophotometer (Agilent Technologies Cary 300) and a Spectrofluorometer (PerkinElmer LS55), respectively. All compounds are > 95% pure by HPLC analysis. Reverse phase HPLC was performed on an Agilent HPLC 1100 using ZORBAX Eclipse XDB-C18 (4.6 mm × 150 mm, 5 μm ID) column. The mobile phase contained 50% 20 mM ammonium acetate and 50% acetonitrile, which was run isocratically. The flow rate was 1 ml/min. The analysis was monitored by a UV-Vis detector at a wavelength of 280 nm.

#### 2.1.1 4-{2-[2-(2-azidoethoxy)ethoxy]ethoxy}-4-oxobutanoic acid (1)

2-[2-(2-Azidoethoxy)ethoxy]ethanol (1.1848 g, 6.7666 mmol) and succinic anhydride (3.0340 g, 30.319 mmol) were mixed in dry DMF (5 ml) and stirred at 55 °C for 24 h. After that, the reaction was extracted with dichloromethane and DI water (3 × 100 ml) and followed by brine (100 ml). The organic layer was dried by anhydrous Na_2_SO_4_ and the solvent was removed under vacuum pressure. The product was obtained without purification to yield 1.8016 g (95%) of **1** as a pale-yellow oil. ^1^H NMR (500 MHz, DMSO-d_6_): δ = 12.20 (s, 1H), 4.21 (t, *J* = 5.0 Hz, 2H), 3.69 (t, *J* = 5.0 Hz, 2H), 3.64 (m, 2H), 3.46 (t, *J* = 5.0 Hz, 2H), 2.97 (s, 2H), 2.81 (s, 2H), 2.58 (m, 2H), 2.55 (m, 2H). ^13^C NMR (125 MHz, DMSO-d_6_): δ = 174.0, 172.6, 70.3, 70.1, 69.4, 68.8, 63.8, 50.5, 31.2, 29.2 ppm. MS (high resolution ESI+) m/z: the calculated value (calcd) for C_10_H_17_N_3_NaO_6_ ([M + Na]^+^): 298.1015, found 298.1010.

#### 2.1.2 2-[2-(2-azidoethoxy)ethoxy]ethyl 4-oxo-4-[(5-sulfamoyl-1,3,4-thiadiazol-2-yl)amino]butanoate (2)

**1** (1.5611 g, 5.6744 mmol) and hydrolyzed acetazolamide (1.1235 g, 6.2417 mmol) were dissolved in dry DMF (5 ml). The reaction mixture was then cooled to 0 °C. 1-Ethyl-3-(3-dimethylaminopropyl)carbodiimide (EDC, 1.6317 g, 8.5117 mmol) and 4-dimethylaminopyridine (DMAP, 0.1386 g, 1.134 mmol) were added into the mixture at 0 °C. The reaction mixture was warm up to 30 °C and stirred for 24 h. After that, the reaction was extracted with HCl (0.2 N, 1 × 100 ml), DI water (2 × 100 ml) and brine (1 × 100 ml), respectively. The organic layer was then dried over anhydrous Na_2_SO_4_ and the solvent was removed under reduced pressure. The obtained residue was purified by silica chromatography eluting with hexane: ethyl acetate (3: 1 to 2: 1) to yield 1.2547 g (50%) of **2** as a pale yellow oil. ^1^H NMR (500 MHz, DMSO-d_6_): δ = 8.09 (s, 1H), 7.00 (s, 2H), 4.32 (t, *J* = 5.0 Hz, 2H), 3.78 (t, *J* = 5.0 Hz, 2H), 3.74 (m, 2H), 3.47 (t, *J* = 5.0 Hz, 2H), 3.04 (s, 1H), 3.00 (t, *J* = 7.0 Hz, 2H), 2.95 (s, 1H), 2.86 (t, *J* = 7.0 Hz, 2H). ^13^C NMR (125 MHz, DMSO-d_6_): δ = 172.8, 171.0, 164.4, 162.9, 70.5, 70.4, 69.9, 69.0, 64.2, 50.7 ppm. MS (high resolution ESI+) m/z: the calculated value (calcd) for C_12_H_19_N_7_NaO_7_S_2_ ([M + Na]^+^): 460.0685, found 460.0680.

#### 2.1.3 *N,N'*-((5,5-difluoro-3,7-bis(4-methoxyphenyl)-5H-4l4,5l4-dipyrrolo[1,2-c:2′,1′-f][1,3,5,2]triazaborinine-1,9-diyl)bis(3,1-phenylene))bis(hex-5-ynamide) (3)

AZB-NH_2_ (0.2040 g, 0.3468 mmol) and 5-hexynoic acid (0.20 ml, ρ = 1.03, 1.8 mmol) were dissolved in dry dichloromethane (6 ml). The reaction mixture was then cooled to 0 °C. 1-Ethyl-3-(3-dimethylaminopropyl)carbodiimide (EDC, 0.2600 g, 1.3563 mmol) and 4-dimethylaminopyridine (DMAP, 0.0350 g, 0.0286 mmol) were added into the mixture at 0°C. The reaction mixture was warm up to 30°C and stirred for 2 h. After that, the reaction was extracted with DI water (3 × 100 ml) and brine (1 × 100 ml), respectively. The organic layer was then dried over anhydrous Na_2_SO_4_ and the solvent was removed under reduced pressure. The obtained residue was purified by silica chromatography eluting with dichloromethane: MeOH (100: 0 to 98: 2) to yield 0.1972 g (73%) of **3** as a red metallic solid. ^1^H NMR (500 MHz, CDCl_3_ + 3 drops of MeOD-d_4_): δ = 7.97 (d, *J* = 9.0 Hz, 2H), 7.95 (s, 1H), 7.67 (s, 1H), 7.66 (s, 1H), 7.28 (t, *J* = 6.0 Hz, 1H), 6.95 (s, 1H), 6.92 (d, *J* = 9.0 Hz, 2H), 3.82 (s, 1H), 2.39 (t, *J* = 7.5 Hz, 2H), 2.25 (t, *J* = 7.5 Hz, 2H), 1.97 (s, 1H), 1.85 (t, *J* = 7.5 Hz, 2H). ^13^C NMR (125 MHz, CDCl_3_ + 3 drops of MeOD-d_4_): δ = 171.4, 162.0, 145.2, 142.3, 138.4, 133.0, 131.7, 129.0, 125.0, 123.9, 120.7, 119.0, 114.9, 83.6, 69.3, 55.4, 35.7, 24.0, 17.9 ppm. MS (high resolution ESI^+^) m/z: the calculated value (calcd) for C_46_H_40_BF_2_N_5_NaO_4_ ([M + Na]^+^): 798.3039, found 798.3041.

#### 2.1.4 N,N'-((5,5-difluoro-2-iodo-3,7-bis(4-methoxyphenyl)-5H-5l4,6l4-dipyrrolo[1,2-c:2′,1′-f][1,3,5,2]triazaborinine-1,9-diyl)bis(3,1-phenylene))bis(hex-5-ynamide) (4)

**3** (97.7 mg, 0.1260 mmol) was dissolved in 6 ml of CHCl_3_: CH_3_COOH (3:1). *N*-iodosuccinimide (NIS, 63.2 mg, 0.281 mmol) was then added to the solution and stirred at 30 °C for 1 h. After that, the reaction was stopped by adding DI water (20 ml) and extracted with sat. Na_2_SO_3_ (2 × 20 ml), sat. NaHCO_3_ (2 × 20 ml) and brine (1 × 100 ml), respectively. The organic layer was then dried over anhydrous Na_2_SO_4_ and the solvent was removed under reduced pressure. The obtained residue was purified by silica chromatography eluting with 100% dichloromethane to yield 54.5 mg (48%) of **4** as a red metallic solid. ^1^H NMR (500 MHz, DMSO-d_6_): δ = 10.21 (s, 1H), 10.07 (d, *J* = 9.0 Hz, 2H), 8.27 (s, 1H), 8.24 (s, 1H), 8.17 (m, 1H), 7.93 (d, *J* = 8.0 Hz, 1H), 7.80 (d, *J* = 7.5 Hz, 1H), 7.72 (m, 2H), 7.69 (m, 1H), 7.53 (m, 1H), 7.38 (t, *J* = 7.5 Hz, 1H), 7.22 (s, 1H), 7.21 (d, *J* = 9.0 Hz, 2H), 7.18 (d, *J* = 9.0 Hz, 2H), 3.95 (s, 3H), 3.94 (s, 3H), 2.91 (d, *J* = 8.5 Hz, 2H), 2.55 (m, 2H), 2.33 (d, *J* = 7.5 Hz, 2H), 1.87 (dd, *J* = 7.0, 5.0 Hz, 2H). ^13^C NMR (125 MHz, DMSO-d_6_): δ = 207.3, 171.2, 163.7. 162.4, 161.0, 155.8, 146.8, 145.6, 143.8, 142.9, 139.9, 139.6, 133.3, 133.2, 132.7, 131.8, 129.3, 128.8, 126.1, 125.2, 124.2, 122.6, 122.1, 121.9, 121.1, 120.4, 119.9, 115.2, 113.8, 84.5, 72.1, 56.2, 55.8, 35.5, 24.4, 17.9 ppm, ^19^F NMR (470 MHz, DMSO-d_6_): δ = -131.23 (q, *J* = 33.0 Hz, BF_2_) ppm. MS (high resolution ESI^+^) m/z: the calculated value (calcd) for C_46_H_39_BF_2_IN_5_NaO_4_ ([M + Na]^+^): 924.2006, found 924.2008.

#### 2.1.5 4,4′-(((((((((((5,5-difluoro-2-iodo-3,7-bis(4-methoxyphenyl)-5H-5l4,6l4-dipyrrolo[1,2-c:2′,1′-f][1,3,5,2]triazaborinine-1,9-diyl)bis(3,1-phenylene))bis(azanediyl))bis(4-oxobutane-4,1-diyl))bis(1H-1,2,3-triazole-4,1-diyl))bis(ethane-2,1-diyl))bis(oxy))bis(ethane-2,1-diyl))bis(oxy))bis(ethane-2,1-diyl))bis(oxy))bis(4-oxobutanoic acid) (AZB-I-Control)

**4** (28.7 mg, 0.0318 mmol) and **1** (38.4 mg, 0.140 mmol) were dissolved in DMSO (0.5 ml). To the reaction mixture was added freshly prepared Na ascorbate solution (1.5 mg in 20 μl of DI water, 7.6 μmol) and freshly prepared CuSO_4_•5H_2_O solution (1.0 mg in 20 μl of DI water, 4.0 μmol), respectively. The resulting mixture was stirred vigorously at 30 °C for 1 h. After that, the reaction was stopped and precipitated by adding DI water (20 ml). Then, the product was obtained by centrifugation (5,000 rpm for 10 min) and washed with DI water (20 ml) for 5 times. The product was obtained without purification to yield 28.1 mg (61%) of AZB-I-Control as a dark green solid. ^1^H NMR (500 MHz, DMSO-d_6_): δ = 12.23 (broad s, 2H), 10.16 (s, 1H), 10.02 (s, 1H), 8.27 (d, *J* = 8.5 Hz, 2H), 8.25 (s, 1H), 8.17 (s, 1H), 7.94 (s, 1H), 7.92 (s, 1H), 7.89 (s, 1H), 7.79 (d, *J* = 8.0 Hz, 1H), 7.68 (d, *J* = 8.5 Hz, 2H), 7.53 (m, 1H), 7.50 (m, 1H), 7.36 (t, *J* = 7.5 Hz, 1H), 7.22 (s, 1H), 7.20 (d, *J* = 8.5 Hz, 2H), 7.18 (d, *J* = 8.5 Hz, 2H), 4.54 (m, 4H), 4.17 (m, 4H), 3.96 (s, 3H), 3.95 (s, 3H), 3.87 (m, 4H), 3.62 (m, 4H), 3.59 (m, 4H), 3.57 (m, 4H), 2.77 (m, 4H), 2.54 (m, 4H), 2.53 (m, 4H), 2.51 (m, 4H), 2.00 (m, 4H). ^13^C NMR (125 MHz, DMSO-d_6_): δ = 173.8, 172.6, 171.6, 163.7, 162.4, 161.0, 156.0, 155.8, 146.8, 145.6, 143.9, 142.9, 140.0, 139.7, 133.3, 133.2, 132.7, 131.8, 129.3, 128.8, 126.1, 125.2, 124.2, 122.8, 122.1, 121.8, 121.1, 120.4, 119.8, 115.4, 115.2, 113.8, 83.1, 70.1, 70.0, 69.2, 68.7, 63.8, 56.2, 55.8, 49.9, 36.3, 29.1, 29.0, 25.3, 25.1 ppm, ^19^F NMR (470 MHz, DMSO-d_6_): δ = −131.23 (q, *J* = 33.0 Hz, BF_2_) ppm. MS (high resolution ESI^+^) m/z: the calculated value (calcd) for C_66_H_73_BF_2_IN_11_O_16_ ([M]^+^): 1451.4343, found 1451.4348. The compound was 98% pure by HPLC analysis with retention time 3.59 min (Supplementary Figure S1).

#### 2.1.6 2-(2-(2-(4-(4-((3-(5,5-difluoro-2-iodo-3,7-bis(4-methoxyphenyl)-9-(3-(4-(1-(2-(2-(2-((4-oxo-4-((5-sulfamoyl-1,3,4-thiadiazol-2-yl)amino)butanoyl)oxy)ethoxy)ethoxy)ethyl)-1H-1,2,3-triazol-4-yl)butanamido)phenyl)-5H-5l4,6l4-dipyrrolo[1,2-c:2′,1′-f][1,3,5,2]triazaborinin-1-yl)phenyl)amino)-4-oxobutyl)-1H-1,2,3-triazol-1-yl)ethoxy)ethoxy)ethyl 4-oxo-4-((5-sulfamoyl-1,3,4-thiadiazol-2-yl)amino)butanoate (AZB-I-CAIX_2_)

**4** (40.0 mg, 0.0444 mmol) and **2** (60.8 mg, 0.139 mmol) were dissolved in DMSO (0.5 ml). To the reaction mixture was added freshly prepared Na ascorbate solution (3.0 mg in 20 μl of DI water, 15 μmol) and freshly prepared CuSO_4_•5H_2_O solution (2.0 mg in 20 μl of DI water, 8.0 μmol), respectively. The resulting mixture was stirred vigorously at 30 °C for 1 h. After that, the reaction was stopped and precipitated by adding DI water (20 ml). Then, the product was obtained by centrifugation (5,000 rpm for 10 min) and washed with DI water (20 ml) for 5 times. The product was obtained without purification to yield 48.4 mg (61%) of AZB-I-CAIX_2_ as a dark green solid. ^1^H NMR (500 MHz, DMSO-d_6_): δ = 10.07 (s, 1H), 9.93 (s, 1H), 8.30 (s, 4H), 8.19 (d, *J* = 8.5 Hz, 2H), 8.16 (s, 1H), 8.09 (s, 1H), 7.86 (m, 1H), 7.85 (m, 1H), 7.84 (m, 1H), 7.70 (d, *J* = 7.5 Hz, 2H), 7.62 (s, 1H), 7.60 (d, *J* = 8.5 Hz, 2H), 7.45 (m, 1H), 7.42 (m, 1H), 7.29 (t, *J* = 7.5 Hz, 1H), 7.28 (s, 1H), 7.13 (d, *J* = 8.5 Hz, 2H), 7.10 (d, *J* = 8.5 Hz, 2H), 4.46 (t, *J* = 6.0 Hz, 4H), 4.09 (t, *J* = 4.0 Hz, 4H), 3.88 (s, 3H), 3.87 (s, 3H), 3.79 (t, *J* = 6.0 Hz, 4H), 3.54 (t, *J* = 4.0 Hz, 4H), 3.49 (m, 4H), 3.48 (m, 4H), 2.80 (m, 4H), 2.69 (m, 4H), 2.68 (t, *J* = 6.5 Hz, 4H), 2.42 (m, 4H), 1.94 (m, 4H). ^13^C NMR (125 MHz, DMSO-d_6_): δ = 171.6, 171.5, 171.4, 171.2, 171.1, 163.7, 161.0, 158.0, 157.0, 155.9, 146.8, 145.6, 142.9, 140.0, 139.6, 133.3, 133.2, 132.7, 131.8, 129.3, 128.8, 126.1, 125.2, 124.3, 122.7, 122.6, 122.1, 121.9, 121.1, 120.4, 120.0, 115.2, 114.9, 113.8, 72.8, 72.1, 70.1, 69.2, 60.7, 56.2, 55.8, 49.7, 36.3, 35.6, 25.4, 25.1, 24.4 ppm. 19F NMR (470 MHz, DMSO-d_6_): δ = −131.22 (q, *J* = 33.0 Hz, BF2) ppm. MS (high resolution ESI^−^) m/z: the calculated value (calcd) for C_70_H_76_BF_2_IN_19_O_18_S_4_ ([M-H]^-^): 1774.3599, found 1774.3587. The compound was 96% pure by HPLC analysis with a retention time of 11.51 min (Supplementary Figure S1).

### 2.2 Fluorescent quantum yields

The UV/vis absorption spectra and fluorescence were recorded on a UV-vis Spectrophotometer (Agilent Technologies Cary 300) and a spectrofluorometer (PerkinElmer LS55), respectively. Briefly, AZB-I-CAIX_2_ and AZB-I-Control are stocked in DMSO. The stock solutions of AZB-I-CAIX_2_ and AZB-I-Control were added to a quartz cell of 1 cm path length in various solvents (CHCl_3_, DMSO, MeOH, and PBS containing 3% tween80) to a final concentration of 1 μM. The fluorescence spectra were measured at excitation wavelengths of 670 nm. The fluorescence quantum yields were calculated using Eq. 1,

$$\Phi_{f}=\Phi_{std}\left(\frac{A_{sample}}{A_{std}}\right)\left(\frac{I_{std}}{I_{sample}}\right){\left(\frac{\eta_{sample}}{\eta_{std}}\right)}^{2},$$ (1)

where Φ_std_ denotes the fluorescence quantum yield of standard Zn-phthalocyanine in pyridine, A is the peak area of emission, I is the absorbance at the excitation wavelength, and ŋ stands for the solvent reflective index.

### 2.3 Singlet oxygen quantum yields

Singlet oxygen quantum yields of AZB-I-CAIX_2_ and AZB-I-Control were determined using a red LED lamp (660 nm, power density of 8.7 mW cm^−2^) in DMSO at room temperature compared to a standard singlet oxygen probe (methylene blue). A solution of DMSO containing 50 μM of 1,3-diphenylisobenzofuran (DPBF) as a singlet oxygen scavenger and 0.5 μM of AZB-I-CAIX_2_ in a quartz cell of 1 cm path length. The DPBF solution in DMSO (negative control), the solution containing 0.5 µM of AZB-I-Control, and the solution containing 0.5 µM methylene blue (comparative control) were also examined. After being exposed to the lamp, the absorbance reduction of DPBF at 408 nm was measured during 0–60 s by an Agilent UV-Vis spectrophotometer (carry 300). The changing of absorbance is plotted against irradiation time. The singlet oxygen quantum yield was calculated according to Eq. 2,

$$\Phi_{\Delta }=\Phi_{std}\left(\frac{{grad}_{sample}}{{grad}_{std}}\right)\left(\frac{F_{std}}{F_{sample}}\right),$$ (2)

where Φ_std_ denotes the singlet oxygen quantum yield of methylene blue (0.52 in DMSO), grad is the rate of reaction, and F is the absorption correction factor (F = 1–10^−absorbance^).

### 2.4 Biological studies experiments

#### 2.4.1 Cell cultures

For human cell lines, CAIX expression in MDA-MB-231 (human breast cancer) is significantly higher than in other cell lines including MCF-7 (human breast cancer), HeLa (human cervical cancer), A549 (human lung cancer), and HEK293 (human embryonic kidney). Therefore, those cell lines are performed using different endogenous CAIX expression levels. MDA-MB-231, MCF-7, HeLa, and HEK293 were cultured in Dulbecco’s Modified Eagle’s Media (DMEM, Gibco) and A549 in Kaighn’s Modification of Ham’s F-12 Medium (F-12K, ATCC). For murine cell lines, 4T1 (murine mammary carcinoma) is higher CAIX expression than 67NR (mouse breast cancer). Two murine cell lines were cultured in RPMI (Gibco). All cell lines were cultured on 75 cm^3^ cell culture flasks (NEST) supplemented with 10% fetal bovine serum (FBS, Hyclone) and 1% penicillin-streptomycin (P/S, Corning) under humidified 95% air, 5% CO_2_ atmosphere at 37 °C.

#### 2.4.2 Time and dose dependent cellular uptake in human cell lines

All human cell lines (MDA-MB-231, MCF-7, HeLa, A549, and HEK293) were seeded on 8-well (LabTek II Cover RS Glass Slide Sterile) at 1 × 10^4^/well and incubated at 37 °C for 24 h. After that, the cells were incubated with 5 μM of AZB-I-CAIX_2_ (all cells) and AZB-I-Control (MDA-MB-231 and MCF-7) for 0, 1, 3, 6, and 24 h. For dose-dependent experiment, MDA-MB-231 cells were incubated with 0, 1, 2.5, and 5 μM of AZB-I-CAIX_2_ for 0 and 6 h. Then, the cells were washed three times with 0.01 M of PBS buffer (pH 7.4) and treated with medium containing 1.0 µM Hoechst 33,342 (DNA fluorescent staining, Thermo Fisher Scientific) for 10 min. The cells were visualized under ×60 oil immersion objective lens by Laser Scanning Confocal Microscope (Nikon A1Rsi) with 561 nm laser (AZB-I-CAIX_2_ and AZB-I-Control) and 405 nm laser (Hoechst33342). Quantitative corrected total cell fluorescence data was quantified using ImageJ and represented the mean ± SD (*n* = 50). Statistical analysis: One-way ANOVA followed by Tukey’s analysis was used for comparison between multiple groups using GraphPad Prism9 software. *p* values of less than 0.05 (95% confidence interval) are considered significant (ns *p* < 0.12, * *p* < 0.033, ** *p* < 0.002, *** *p* < 0.001).

#### 2.4.3 Co-cultured between (+) and (−) carbonic anhydrase IX expression cell lines

Positive CAIX expression cell (MDA-MB-231) and negative CAIX expression cell (MCF-7) were seeded on 35-mm glass-bottom confocal dishes (NEST) at 2 × 10^5^/well in single media and incubated at 37 °C for 24 h. After that, the cells were incubated with 5 μM of AZB-I-CAIX_2_ for 6 h. Then, the cells were washed three times with 0.01 M of PBS buffer (pH 7.4) and treated with medium containing 1.0 µM Hoechst33342 for 10 min. The cells were visualized under ×60 oil immersion objective lens by Laser Scanning Confocal Microscope (Nikon A1Rsi) with 561 nm laser (AZB-I-CAIX_2_) and 405 nm laser (Hoechst33342).

#### 2.4.4 Competitive effect with carbonic anhydrase IX ligand

Positive CAIX expression cell lines (MDA-MB-231) were seeded on 8-well (LabTek II Chamber Slide x/Cover RS Glass Slide Sterile) at 1 × 10^4^/well and incubated at 37°C for 24 h. After that, the cells were incubated with 5 μM of AZB-I-CAIX_2_ and 0, 5, 50, 100, 500, and 1,000 μM of acetazolamide (CAIX ligand) for 6 h. For time-dependent competition effect with CAIX inhibitor, MDA-MB-231 cells were incubated with 5 μM of AZB-I-CAIX_2_ and 500 μM of acetazolamide for 0 h, 1 h, 3 h, and 6 h. Then, the cells were washed three times with 0.01 M of PBS buffer (pH 7.4) and treated with a medium containing 1.0 µM Hoechst33342 for 10 min. The cells were visualized under ×60 oil immersion objective lens by Laser Scanning Confocal Microscope (Nikon A1Rsi) with 561 nm laser (AZB-I-CAIX_2_) and 405 nm laser (Hoechst33342). Quantitative corrected total cell fluorescence data was quantified using ImageJ and represented the mean ± SD (*n* = 50). Statistical analysis: t-test followed by Tukey’s analysis was used for comparison between multiple groups using GraphPad Prism9 software. *p* values of less than 0.05 (95% confidence interval) are considered significant (ns *p* < 0.12, * *p* < 0.033, ** *p* < 0.002, *** *p* < 0.001).

#### 2.4.5 Colocalization study

Positive CAIX expression cell lines (MDA-MB-231) were seeded on 8-well (LabTek II Chamber Slide x/Cover RS Glass Slide Sterile) at 1 × 10^4^/well and incubated at 37°C for 24 h. After that, the cells were incubated with 5 μM of AZB-I-CAIX_2_ for 6 h. Then, the cells were washed three times with 0.01 M of PBS buffer (pH 7.4) and treated with 1.0 µM Hoechst33342 in medium containing 1.0 µM of MitoTracker™ Green FM (Thermo Fisher Scientific), LysoTracker™ Green DND-26 (Thermo Fisher Scientific), C6-NBD Ceramide (Golgi tracker, Avanti Polar Lipids), and ER-Tracker™ Green (BODIPY™ FL Glibenclamide, Thermo Fisher Scientific) for 20 min. The cells were visualized under ×60 oil immersion objective lens by Laser Scanning Confocal Microscope (Nikon A1Rsi) with 641 nm laser (AZB-I-CAIX_2_), 488 nm laser (Mitotracker, Lysotracker, Golgi tracker, and ER tracker), and 405 nm laser (Hoechst33342). Pearson’s correlation coefficient for colocalization of AZB-I-CAIX_2_ and organelles trackers were obtained from ImageJ

#### 2.4.6 Cell cytotoxicity assay

All human cell lines (MDA-MB-231, MCF-7, HeLa, A549, and HEK293) were seeded on a 96-well cell culture plate at approximately 7 × 10^3^ cells per well for 24 h. After that, the cells were incubated with 0, 0.125, 0.25, 0.5, 1, 2, 5, and 10 μM of AZB-I-CAIX_2_ (all cells) and AZB-I-Control (MDA-MB-231 and MCF-7) for 6 h. After incubation, the cells were washed with 0.01 M of PBS pH 7.4 (3 times) before being irradiated by a red LED lamp (660 nm, power density of 8.7 mW cm^−2^) for 0 min, 5 min, 10 min, and 15 min, and then re-incubated for another 24 h. Then, the cells were added with 0.5 mg ml^−1^ of MTT reagent (Methylthiazolyldiphenyl-tetrazolium bromide, Sigma-Aldrich) in 0.01 M PBS (pH 7.4) solution for 2.5 h. After the solution of MTT reagent removal, the formazan product was dissolved by adding DMSO. The cell cytotoxicity was detected through UV-vis absorption of formazan at wavelength 560 nm using a microplate reader (BMG Labtech/SPECTROstar Nano). IC_50_ values of cell viability were evaluated by GraphPad Prism9 software. Statistical analysis: One-way ANOVA followed by Tukey’s analysis was used for comparison between multiple groups using GraphPad Prism9 software. *p* values of less than 0.05 (95% confidence interval) are considered significant (ns *p* < 0.12, * *p* 0.033, ** *p* < 0.002, *** *p* < 0.001).

#### 2.4.7 Live/dead staining

Positive CAIX expression cells (MDA-MB-231) were seeded on a 6-well cell culture plate at 2 × 10^5^/well and incubated at 37 °C for 24 h. After that, the cells were incubated with 0.5 μM of AZB-I-CAIX_2_ for 6 h. Then, the cells were washed three times with 0.01 M of PBS buffer (pH 7.4) After incubation, the cells were irradiated by a red LED lamp (660 nm, power density of 8.7 mW cm^−2^) for 10 min before re-incubation for another 24 h. Thereafter, the cells were stained with 4 µM calcein-AM and propidium iodide (PI) (Thermo Fisher Scientific) for 5 min, and then imaged by Fluorescence microscope (BioRad/Zoe) using λ_ex_ = 490 nm and λ_em_ = 515 nm for calcein AM and λ_ex_ = 535 nm and λ_em_ = 615 nm for PI.

#### 2.4.8 Intracellular singlet oxygen generation

Positive CAIX expression cell line (MDA-MB-231) was seeded on 8-well chambered coverglass (LabTek, Nunc) at 1 × 10^4^/well and incubated at 37°C for 24 h. After that, the cells were incubated with 0.125 and 0.25 μM of AZB-I-CAIX_2_ for 6 h. Then, the cells were washed three times with 0.01 M of PBS buffer (pH 7.4). Thereafter, 20 µM of 2,7-dichloro-dihydro-fluorescein diacetate (DCFH-DA, Sigma-Aldrich) was incubated in the cells for 1 h. The cells were washed three times with 0.01 M of PBS buffer (pH 7.4) and irradiated with a red LED lamp (660 nm, power density of 8.7 mW cm^−2^) for 10 min. Before imaging, the cells were treated with medium containing 1.0 µM Hoechst33342 for 10 min. The cells were visualized under ×60 oil immersion objective lens by Laser Scanning Confocal Microscope (Nikon A1Rsi) with 561 nm laser (AZB-I-CAIX_2_) and 405 nm laser (Hoechst33342).

#### 2.4.9 Apoptosis detection

Approximately 1 × 10^6^ cells/well of MDA-MB-231 were seeded on a 6-well plate for 24 h. After that, the cells were treated with 0.5 μM of AZB-I-CAIX_2_ and 1 mM of ROS scavenger reagent (*N*-acetyl-*L*-cysteine (NAC, TCI) for 6 h. The cells were then washed with 0.01 M PBS buffer (pH 7.4) twice before irradiation with a red LED lamp (660 nm, power density of 8.7 mW cm^−2^) for 5 min. Then, the cells were trypsinized and washed three times with ice-cold PBS buffer (0.01 M, pH 7.4) by centrifugation at 4,000 rpm at 4°C for 3 min and resuspend in 1X Annexin binding buffer (Thermo Fisher Scientific). The cells were incubated on ice with Annexin V fluorescein conjugate (FITC annexin V, Thermo Fisher Scientific) at room temperature for 15 min before adding propidium iodide (PI, Thermo Fisher Scientific). Then, 1 × 10^4^ events were analysed by flow cytometry using an Attune NxT Flow Cytometer (Thermo Fisher Scientific).

#### 2.4.10 Murine cell internalization under hypoxia condition

Murine cell lines (4T1 and 67NR) were seeded on 8-well chambered coverglass (LabTek, Nunc) at 1 × 10^4^/well and incubated at 37 °C for 24 h. After that, the cells were incubated under normoxia (humidified 95% air, 5% CO_2_ atmosphere) and hypoxia (5% pO_2_) conditions for 12 h. Then, the cells were treated with 5 μM of AZB-I-CAIX_2_ in DMEM for 0, 1, 3, and 6 h. Thereafter, the cells were washed three times with 0.01 M PBS buffer (pH 7.4) and treated with medium containing 1.0 µM Hoechst33342 for 10 min. Confocal images were visualized under ×60 oil immersion objective lens by Laser Scanning Confocal Microscope (Nikon A1Rsi) with 561 nm laser (AZB-I-CAIX_2_) and 405 nm laser (Hoechst33342).

#### 2.4.11 Murine cell cytotoxicity assay under hypoxia condition

4T1 has seeded on a 96-well plate of approximately 7 × 10^3^ cells per well and incubated in completed medium for 24 h. Thereafter, the cells were incubated under normoxic (humidified 95% air, 5% CO_2_ atmosphere) and hypoxic (5% pO_2_) conditions at 37°C for 12 h before being treated with 0, 0.125. 0.25, 0.5, 5, 10 μM of AZB-I-CAIX_2_ for 6 h. We induced the hypoxia condition following STEMCELL Technologies Inc. (Vancouver, BC, Canada) methods using a hypoxia incubator chamber and references’ protocol (Su et al., 2016). After incubation, the cells were washed twice with 0.01 M of PBS buffer (pH 7.4) to remove excess probes and added to the completed medium before being irradiated with a red LED lamp (660 nm, power density of 8.7 mW cm^−2^) for 0 min, 5 min, 10 min, and 15 min and continued culturing for 24 h in dark. Cell viability was detected by 20 μl of methylthiazolyldiphenyl-tetrazolium bromide (MTT reagent, 0.5 mg ml^−1^, Sigma-Aldrich) for 2.5 h incubation time. After media removal, DMSO was added to dissolve the formazan product and detected through UV-vis absorption of formazan at wavelength 560 nm (BMG Labtech/SPECTROstar Nano microplate reader). IC_50_ values of cell viability were evaluated by GraphPad Prism9 software. Statistical analysis: t-test followed using GraphPad Prism9 software. *p* values of less than 0.05 (95% confidence interval) are considered significant (ns *p* < 0.12, * *p* < 0.033, ** *p* < 0.002, *** *p* < 0.001).

#### 2.4.12 Animal model

Female, 6–8 weeks old wild-type Balb/C mice were purchased from the Animal Experimental Unit, University of Malaya, Malaysia. The mice were maintained in the animal facility at Management and Science University, and all experimental procedures were performed accordingly to the protocol approved by the University Ethics Committee of Management and Science University (Approval code: MSU-RMC-02/FR01/08/L3/001).

#### 2.4.13 *In vivo* acute toxicity study

The acute toxicity study on healthy mice (*n* = 2) was assessed through intravenous administration of 30 mg/kg AZB-I-CAIX_2_, 24.60 mg/kg AZB-I-Control (Equiv. to 30 mg/kg AZB-I-CAIX_2_), and 12.5 mg/kg acetazolamide (Equiv. to 100 mg/kg AZB-I-CAIX_2_). The toxicity signs were observed based on the Berlin test of typical symptoms including bodyweight loss, ruffled hair, and behavioural changes for 14 days.

#### 2.4.14 *In vivo* photodynamic Therapy

The fur of Balb/C mice was shaved and murine 4T1 breast carcinoma cell lines at a density of 5 × 10^5^ cells/mouse were orthotopically injected into the mice’s mammary fat pads. AZB-I-CAIX_2_, AZB-I-Control, and acetazolamide were dissolved respectively in a cocktail of 2.5% cremophore EL and 2.5% ethanol, and further resuspended in the saline to a volume of 0.2 ml, for injection. The 4T1 breast tumor growth was observed and measured daily until the tumor size reached 150–180 mm^3^ (for high CAIX expression) (Rabbani et al., 2009). Once the tumor growth reached 150–180 mm^3^, the mice were randomly divided into groups for the experiment. The 4T1 tumor-bearing Balb/c mice were treated intravenously with saline, AZB-I-CAIX_2_ (30 mg/kg), AZB-I-Control (24.60 mg/kg equiv. to 30 mg/kg AZB-I-CAIX_2_), and acetazolamide (3.75 mg/kg equiv. to 30 mg/kg AZB-I-CAIX_2_). The mice were then kept in the dark for 1 h. After 1-h administration, the mice were administered with an anesthesia cocktail of 0.1 ml ketamine (90 mg/kg ketamine and 10 mg/kg xylazine) intraperitoneally. The mice were irradiated by using Lumacare LC-122A fiber-optic light delivery system at 80 J for 8 min (Fluence rate: 160 mW). The tumor size was then observed and measured every 2 days using a digital caliper. Tumor volume was calculated using the formula [(L x W^2^)/2], where the reading for L is the longest dimension and W is the shortest dimension.

#### 2.4.15 Tumor tissue H&E staining

The harvested tissues and organs were fixed in 10% neutral buffered formalin followed by dehydration in ascending concentrations of 70%, 90%, and 100% ethanol. The dehydrated tissues and organs were cleared in xylene and embedded in the paraffin. The tissue blocks were cut (5 µm thickness) and stained with Hematoxylin and Eosin following the procedure as established (Kampaengsri et al., 2022).

#### 2.4.16 Statistical analysis for *in vivo*

The result was analyzed using SPSS (IBM version 21). For *in vivo* analysis, one-way ANOVA (Dunnett’s test) was used for multi-group comparison. A *p*-value less than 0.05 (*p* < 0.05) indicates statistically significant.

## 3 Results and discussion

### 3.1 Synthesis of AZB-I-CAIX_2_ and AZB-I-control

**SCHEME 1:**
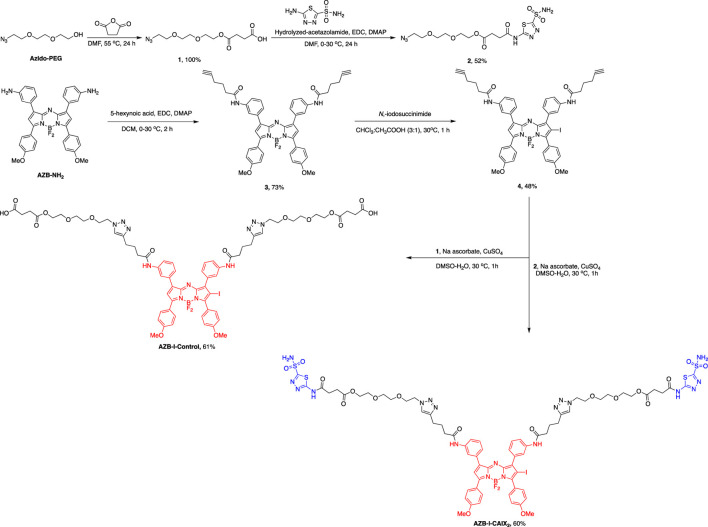
Synthetic scheme of AZB-I-CAIX_2_ and AZB-I-control.

AZB-I-CAIX_2_ was synthesized *via* the azide-alkyne Huisgen cycloaddition between two AZ moieties (**2**) and terminal alkynes of an iodo-aza-BODIPY derivative (**4**), prepared by amide coupling between amino aza-BODIPY (AZB-NH_2_) (Kamkaew and Burgess, 2015) and 5-hexynoic acids followed by mono-iodination. AZB-I-control was produced by a similar reaction condition, except for the linker without AZ (**1**) was used (Scheme 1 and ESI).

In general, when heavy atoms are present in the dye structure, *i.e.* aza-BODIPY, the fluorescent emission is usually quenched due to an increase in the rate of triplet formation that affects orbital spin interaction, the so-called heavy atom effect (De Simone et al., 2017). However, this feature increases singlet oxygen generation which is beneficial for PDT (Kamkaew et al., 2013). As a result, aza-BODIPY was mono iodinated in this design to ensure that the fluorescent signal is preserved while the singlet oxygen yield is sufficient for PDT efficiency.

### 3.2 Photophysical properties of AZB-I-CAIX_2_ and AZB-I-control

**FIGURE 2:**
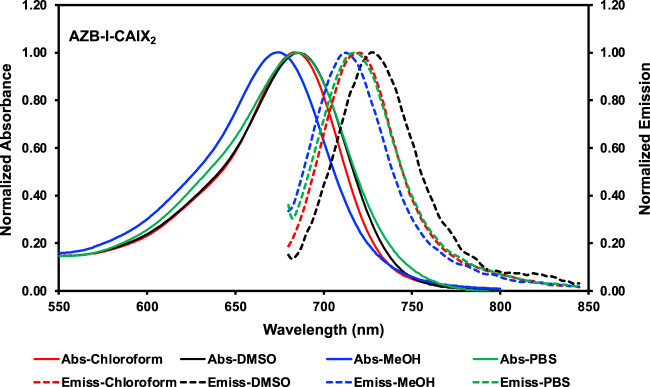
Normalized Vis-NIR absorption and emission spectra of AZB-I-CAIX_2_ in various solvents.

The photophysical properties of AZB-I-CAIX_2_ and AZB-I-control were investigated by UV-VIS-NIR and fluorescence spectrophotometry in various solvents, as shown in Table 1; Figure 2, and Supplementary Figure S2. According to the absorbance spectra, AZB-I-CAIX_2_ and AZB-I-control show similar absorption maxima from 675 to 686 nm in chloroform, DMSO, methanol, and phosphate buffer saline (PBS). The emission maxima of AZB-I-CAIX_2_ are in the range of 713–728 nm in the tested solvents, with fluorescent quantum yields ranging from 0.02–0.09 (Table 1). Interestingly, the emission spectra are red-shifted in the polar aprotic solvents (chloroform and DMSO) when compared to the compound in the polar protic solvents (MeOH and PBS). Similar photophysical properties are observed in the case of AZB-I-control (Supplementary Figure S2 and Table 1).

**TABLE 1:** Photophysical properties of AZB-I-CAIX_2_ and AZB-I-control (1 μM).

Compound	Solvent	λ_max_ (nm)	ε (M^−1^cm^−1^)	λ_emiss_ ^a^ (nm)	∆λ (nm)	Φ_f_ ^b^
AZB-I-CAIX_2_	CHCl_3_	683	11.2 × 10^4^	720	37	0.09
	DMSO	686	11.6 × 10^4^	728	42	0.02
	MeOH	675	9.6 × 10^4^	713	38	0.03
	PBS^c^	685	8.9 × 10^4^	718	33	0.05
AZB-I-Control	CHCl_3_	683	6.0 × 10^4^	720	34	0.14
	DMSO	686	6.5 × 10^4^	728	42	0.04
	MeOH	675	6.6 × 10^4^	714	40	0.05
	PBS^c^	686	6.4 × 10^4^	717	31	0.09

^a^ Samples were excited at 670 nm.

^b^ Relative to Zn-phthalocyanine in pyridine (Φ_f_ = 0.30).

^c^ PBS, containing 3% tween-80.

The data show that AZB-I-CAIX_2_ and AZB-I-control have low fluorescent quantum yields according to the heavy atom effect (Kamkaew et al., 2013), therefore, the singlet oxygen quantum yields of both probes were also investigated. Singlet oxygen generation efficiency of AZB-I-CAIX_2_ and AZB-I-control was measured using 1,3-diphenylisobenzofuran (DPBF) as singlet oxygen (^1^O_2_) scavenger. After AZB-I-CAIX_2_ and AZB-I-control were exposed to the light (660 nm, power density 8.7 mW cm^−2^), ^1^O_2_ was produced within a few seconds in an irradiation time-dependent manner, as indicated by the decreasing DPBF absorbance at 408 nm (Supplementary Figures S3, S4). Moreover, AZB-I-CAIX_2_ and AZB-I-control could generate ^1^O_2_ at a faster rate than methylene blue (standard photosensitizer) in DMSO solution. The calculated singlet oxygen quantum yields (Φ_Δ_) of AZB-I-CAIX_2_ and AZB-I-control were 0.88 and 0.83, respectively, relative to methylene blue. Therefore, both probes could be good for PDT agents with moderate imaging properties.

To ensure that the compound does not degrade during *in vivo* experiments, the stability of AZB-I-CAIX_2_ in fetal bovine serum (FBS) was tested for 7 days. UV-vis-NIR absorption was used to analyze the stability of AZB-I-CAIX_2_ to that of AZB-I-Control (Supplementary Figure S5). The absorbance of AZB-I-CAIX_2_ in FBS remained constant for 7 days, whereas the absorbance of AZB-I-Control began to decline after day 4 in both light and dark conditions. Therefore, AZB-I-CAIX_2_ is suitable for use in further applications.

### 3.3 Cell internalization and carbonic anhydrase IX targeting

**FIGURE 3:**
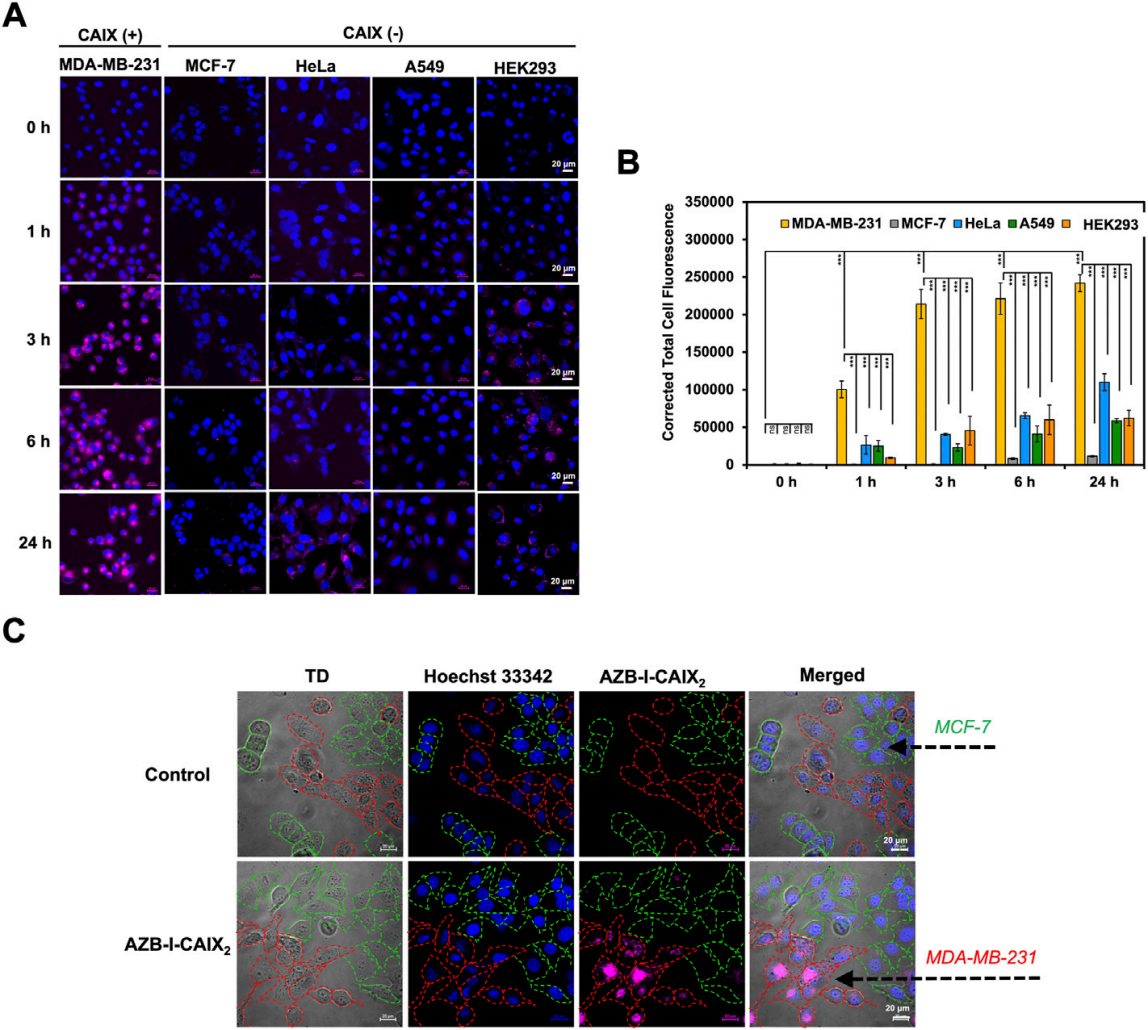
Selective CAIX-dependent uptake of AZB-I-CAIX_2_ on various cells. **(A)** Time-dependent cell internalization of AZB-I-CAIX_2_ tested on CAIX+ (MDA-MB-231) and CAIX- (MCF-7, HeLa, A549, HEK293) cells. **(B)** Corrected Total Cell Fluorescence (CTCF) of signals from experiment **(A) (C)** Confocal images of AZB-I-CAIX_2_ internalized CAIX+ (MDA-MB-231) and CAIX- (MCF-7) in the co-culture system. Statistical analysis: One-way ANOVA followed by Tukey’s analysis was used for comparison between multiple groups using GraphPad Prism9 software. *p* values of less than 0.05 (95% confidence interval) are considered significant (ns *p* < 0.12, * *p* < 0.033, ** *p* < 0.002, *** *p* < 0.001). Scale bar = 20 μm.

To evaluate cancer cell targetability of AZB-I-CAIX_2_, human cancer cell lines with high expression levels of CAIX (MDA-MB-231) (Jung et al., 2017b) and low CAIX levels (MCF-7, HeLa, and A549) (Jung et al., 2017b) were selected for comparison. Moreover, human embryonic kidney cells (HEK293) were also used as normal cell control (Rana et al., 2013). As shown in Figure 3, time-dependent cellular internalization revealed that AZB-I-CAIX_2_ faster internalized and accumulated a higher amount in MDA-MB-231 cells from 1 to 24 h incubation time. Whereas low fluorescent signals of AZB-I-CAIX_2_ were observed from other cell lines even at the longest exposure time, 24 h (Figure 3A). Quantitative fluorescent signals from all cell lines by ImageJ were presented in Figure 3B. In addition, when AZB-I-control was used in the same experiment settings, no observable fluorescent signals were detected from both CAIX positive and negative cells (Supplementary Figure S6). Furthermore, we performed dose-dependent cell internalization in MDA-MB-231 cells and discovered that 5 μM AZB-I-CAIX_2_ provided a clear signal, which will be the best dose for the rest of the *in vitro* tests (Supplementary Figure S7). Additionally, when MDA-MB-231 (CAIX+) and MCF-7 (CAIX–) breast cancer lines were co-cultured and then treated with AZB-I-CAIX_2_, the NIR fluorescent signals were only found in the CAIX+ cells (Figure 3C). These findings revealed that AZB-I-CAIX_2_ had high selectivity for CAIX when it internalized cancer cells.

**FIGURE 4:**
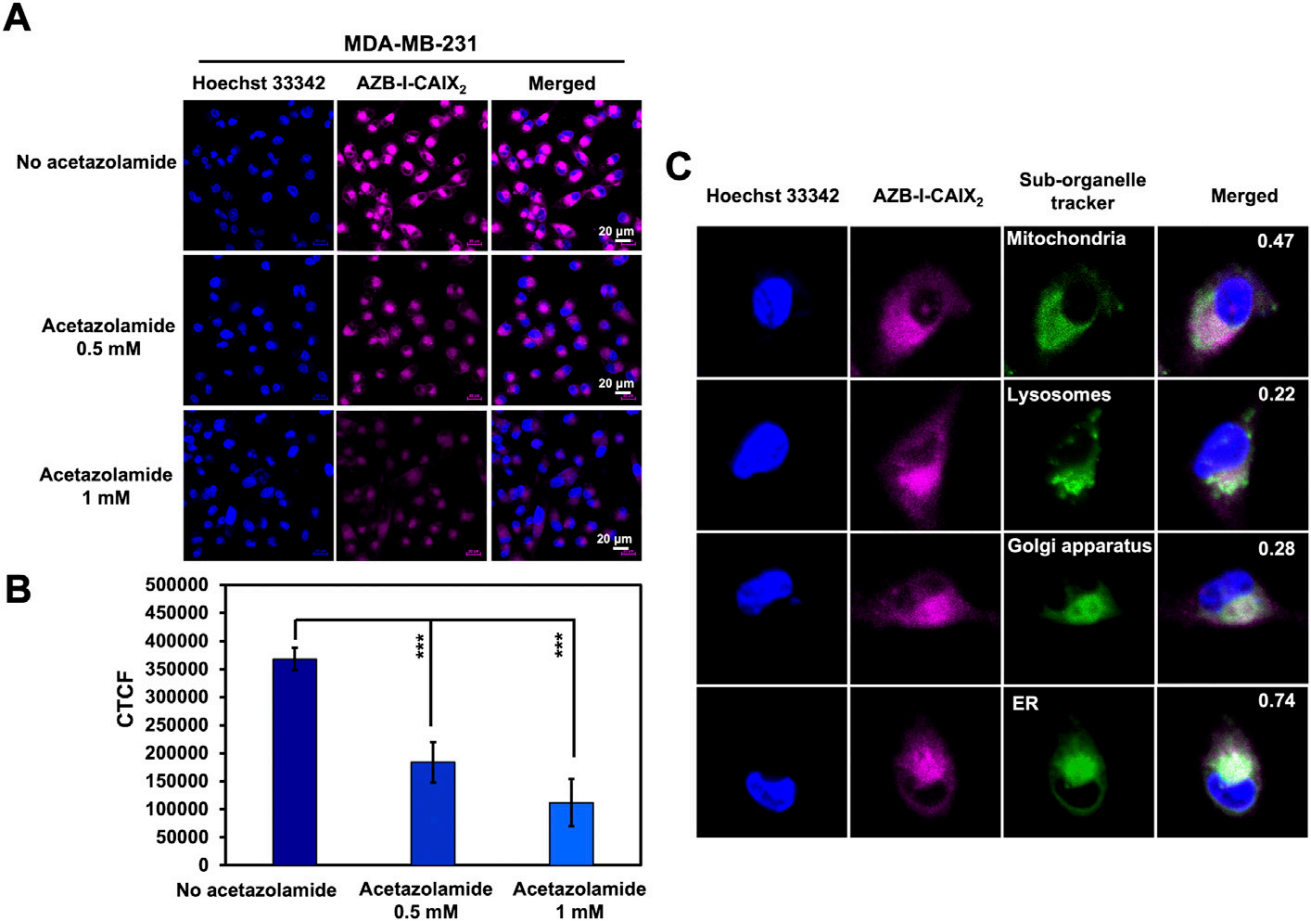
Inhibitory effect of CAIX inhibitor (acetazolamide) on the cellular uptake of AZB-I-CAIX_2_. **(A)** Confocal images of MDA-MB-231 cells incubated with AZB-I-CAIX_2_ (5 μM) in the absence and presence of acetazolamide (0.5 mM and 1.0 mM) for 6 h. **(B)** Corrected Total Cell Fluorescence (CTCF) of signals from experiment **(A) (C)** Colocalization of AZB-I-CAIX_2_ with organelle trackers (MitoTracker, LysoTracker, Golgi and ER markers) in MDA-MB-231 cells with Pearson’s coefficients of 0.47, 0.22, 0.28 and 0.74, respectively. Scale bar = 20 μm.

To further demonstrate the selectivity of AZB-I-CAIX_2_, a competition assay was performed to validate the CAIX-mediated uptake of AZB-I-CAIX_2_ in cancer cells. MDA-MB-231 cells were incubated with AZB-I-CAIX_2_ (5 μM) in the presence of various concentrations (0, 0.5, and 1.0 mM) of CAIX inhibitor, acetazolamide, for 6 h. The cellular uptake of AZB-I-CAIX_2_ was inhibited by a CAIX inhibitor in a dose-dependent manner (Figures 4A,B, and Supplementary Figure S8). In addition, when a shorter time incubation was used in the competitive assay (1 h and 3 h vs. 6 h), the fluorescent signals were reduced in the presence of acetazolamide at every time point (Supplementary Figure S9), confirming that acetazolamide moieties play a key role in cellular internalization.

In addition to cellular uptake, the detected fluorescent signals of AZB-I-CAIX_2_ from MDA-MB-231 cells were found to be colocalized to some degree with MitoTracker, LysoTracker, Golgi, and ER markers with Pearson’s coefficients of 0.47, 0.22, 0.28, and 0.74, respectively (Figure 4C). This confirms that our probe can be localized inside the cancer cells and may indicate the internalization of CAIX in response to AZB-I-CAIX_2_ binding (Hulikova et al., 2009). CAIX internalization has been studied through caveolar-mediated endocytosis (Bourseau-Guilmain et al., 2016) thus suggesting that caveolin-1 protein mediating caveolar-dependent endocytosis might bind to endoplasmic reticulum membrane during endocytic uptake (Schlegel et al., 2001).

### 3.4 *In vitro* photodynamic therapy

**FIGURE 5:**
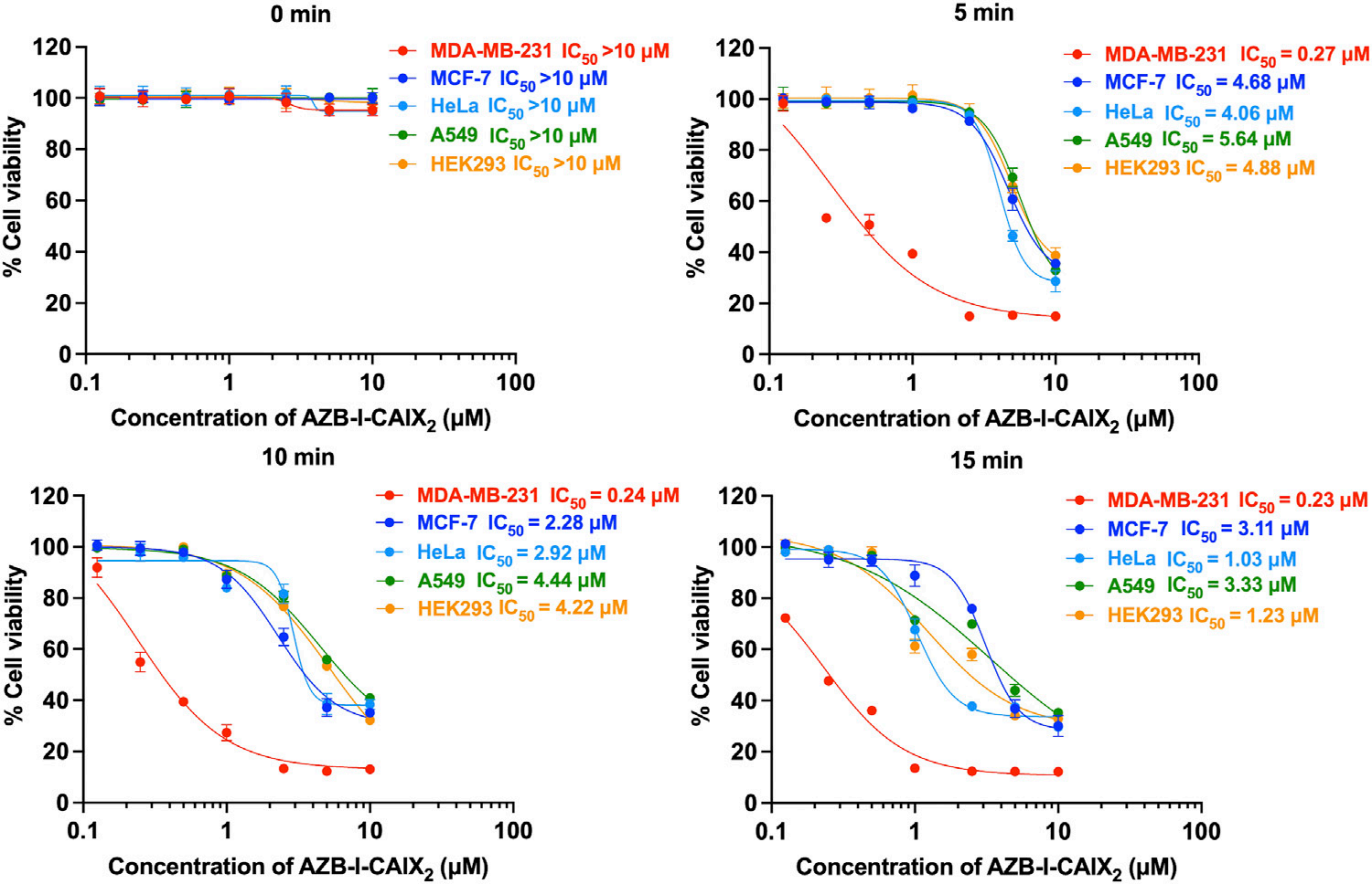
Half maximal inhibitory concentration (IC_50_) curves of AZB-I-CAIX_2_ tested on CAIX+ (MDA-MB-231) and CAIX- (MCF-7, HeLa, A549, HEK293) cells under various light exposure times (0 min, 5 min, 10 min, 15 min). The cells were incubated with various concentrations of AZB-I-CAIX_2_ (0–10 μM) for 6 h and irradiated with a lamp (660 nm, power density of 8.7 mW cm^−2^). IC_50_ was evaluated using GraphPad Prism9 software.

To evaluate the effect of CAIX inhibitor on PDT, assays to measure photocytotoxicity of AZB-I-CAIX_2_ in all cell lines including CAIX+, CAIX- and normal cells were performed. All cells were treated with various doses (0–10 μM) of AZB-I-CAIX_2_ for 6 h before light irradiation (660 nm, power density of 8.7 mW cm^−2^) with different durations (0 min, 5 min, 10 min, and 15 min) and then the cells were cultured in the dark for another 24 h. As shown in Figures 5 and Supplementary Figure S10, AZB-I-CAIX_2_ did not cause cytotoxicity to any cells that were not illuminated (0 min). However, once the irradiation was on, the cell viability of all cells was decreasing as the dose of AZB-I-CAIX_2_ was increased. Remarkably, MDA-MB-231 (CAIX+) is the most sensitive cell to AZB-I-CAIX_2_. After the cells were irradiated for 5 min, the viability of MDA-MB-231 cells was reduced to 50% at a very low concentration with the half-maximal inhibitory concentration (IC_50_) of 0.27 μM. In addition, the probe even causes more photocytotoxicity when irradiation time was longer (10 min and 15 min) in a dose-dependent manner. On the other hand, after 5 min irradiation, all CAIX– including normal cells did not show phototoxicity until the concentration of AZB-I-CAIX_2_ was up to 5 μM. When CAIX + cells were irradiated for only 5 min, IC_50_ values of AZB-I-CAIX_2_ were more than 15 times lower than those of CAIX- cells (Figure 5). However, prolonged light exposure time did not significantly improve the therapeutic index of AZB-I-CAIX_2_ in CAIX + cells. In contrast, the light dose clearly affected cell viability of CAIX- cells.

Furthermore, AZB-I-control (no AZ conjugate) was used to test photo-induced cell toxicity on breast cancer lines (MDA-MD-231 and MCF-7) (Supplementary Figure S11). No dramatic change in cell viability was observed from both cell lines (CAIX+ and CAIX-) even with light irradiation for up to 15 min and compound concentration was up to 10 μM (Supplementary Figure S11). These findings support the importance of AZ moieties in AZB-I-CAIX_2_ for cancer cell uptake through CAIX.

### 3.5 Live/dead cell staining and intracellular reactive oxygen species detection

To further confirm the CAIX+ cancer cells were destroyed by reactive oxygen species (ROS) from light activation reaction, viability/cytotoxicity and intracellular ROS detection assays were performed. Viable and dead cells were visualized using calcein-AM and propidium iodide (PI) staining. Once calcein-AM enters live cells, green fluorescence can be observed after the dye is cleaved by intracellular esterase, while PI only interacts with dead cells nuclei and gives red fluorescence.

**FIGURE 6:**
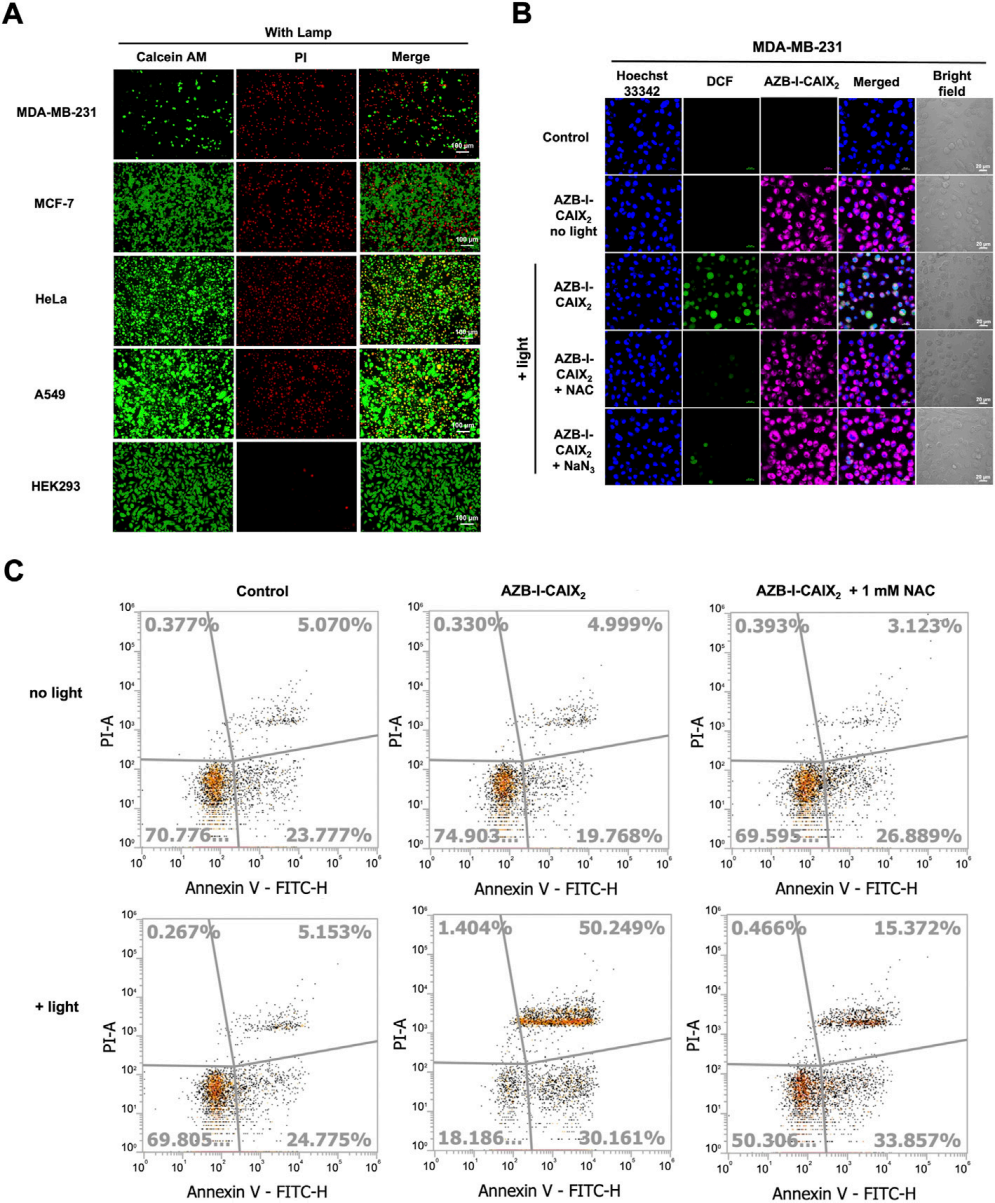
Viability/Cytotoxicity assay and intracellular ROS detection. **(A)** Live/dead cell imaging of CAIX+ (MDA-MB-231) and CAIX- (MCF-7, HeLa, A549, HEK-293) cells incubated with AZB-I-CAIX_2_ (0.5 μM) under 10 min light irradiation. **(B)** Confocal images of MDA-MB-231 cells incubated with AZB-I-CAIX_2_ (0.5 μM) and light irradiation for 10 min in the presence of ROS detection probe, DCFH-DA, and ROS scavengers, NAC or NaN_3_. The green emission signal indicated the existence of ROS inside the cells. **(C)** Flow cytometry Annexin V fluorescein isothiocynate (FITC)/propidium iodide (PI) apoptosis analysis.

As shown in Figure 6A, after cells were incubated with AZB-I-CAIX_2_ followed by light irradiation, a high number of dead cells (indicated as red signal) could be observed from MDA-MB-231 while the live cells (indicated as green signal) were very few. Other CAIX- cancer cells showed some dead cell signals along with a high population of viable cells. Moreover, little to no dead cells were detected in the case of the normal cell (HEK293). In addition, all the cells incubated with AZB-I-CAIX_2_ (no light irradiation) and cells incubated with AZB-I-control (either with or without illumination) mostly remained viable (Supplementary Figure S12).

Subsequent, 2',7′-dichlorofluorescein diacetate (DCFH-DA) was utilized to monitor intracellular ROS generation because its non-fluorescent form can be oxidized by ROS to create the fluorescence 2′,7′-dichlorofluorescein (DCF) that exhibits green fluorescence inside living cells. As shown in Figure 6B, bright green emission is observed only in the case of MDA-MB-231 cells incubated with AZB-I-CAIX_2_ followed by light activation. The green fluorescence increases when a higher amount of AZB-I-CAIX_2_ was used (Supplementary Figure S13), implying the ROS was generated from our probe inside the cells in the dose-response relationship. However, the green emission from DCF was decreased when the ROS scavengers *N*-acetyl-L-cysteine (NAC) (Halasi et al., 2013; Hosseini et al., 2019) and sodium azide (NaN_3_) (Bancirova, 2011) were introduced (Figure 6B). These results demonstrated that a light-activated AZB-I-CAIX_2_ probe was responsible for intracellular ROS production.

For apoptosis detection, phosphatidylserine appearance and DNA fragmentation were used to detect post-PDT cell apoptosis. The apoptosis detection kit, which contains Annexin V fluorescein isothiocynate (FITC) and propidium iodide (PI), was used to identify cells undergoing apoptosis (Lakshmanan and Batra, 2013). When cells were in the early stages of apoptosis, they translocated phosphatidylserine from the inner face of the plasma membrane to the cell surface, where it interacted with Annexin V-FITC to produce green fluorescence (van Engeland et al., 1998) (the cell population indicated in the bottom right of the diagram). Red fluorescence was produced as a result of PI binding to the DNA inside the dead cells (the cell population shown in the top left of the diagram). In the late phases of apoptosis, the integrity of the cell membrane was damaged, allowing Annexin V and PI to enter the cells (the cell population shown in the top right of the diagram). As shown in Figure 6C, the cells treated with AZB-I-CAIX_2_ followed by light irradiation undergo early and late apoptosis (30.2 and 50.3%, respectively), which is significantly higher than cells treated without light (19.8 and 5.0%, respectively). However, when the ROS scavenger, NAC, was introduced, the percentage of late apoptotic MDA-MB-231 cells treated with AZB-I-CAIX_2_-mediated PDT significantly reduced to 15.37%, suggesting that intracellular ROS production plays important role in programmed cell death.

### 3.6 Murine cells study in hypoxia condition

Two murine breast cancer lines were used as a comparison. 4T1 cells are highly metastatic breast cancer cells. Compared to nonmetastatic 67NR cells, tumors formed by 4T1 have significantly higher amounts of hypoxia, necrosis, and apoptosis due to fewer blood vessels (Lou et al., 2011). In addition, the bioinformatics analysis revealed several hypoxia-regulated genes, including CAIX, that are expressed at higher levels in the 4T1 tumors relative to the 67NR (Lou et al., 2008). Therefore, the 4T1 mouse could be a suitable model to study the effect of overexpression of CAIX on the progression of breast cancer (Lou et al., 2011; Chafe et al., 2015).

**FIGURE 7:**
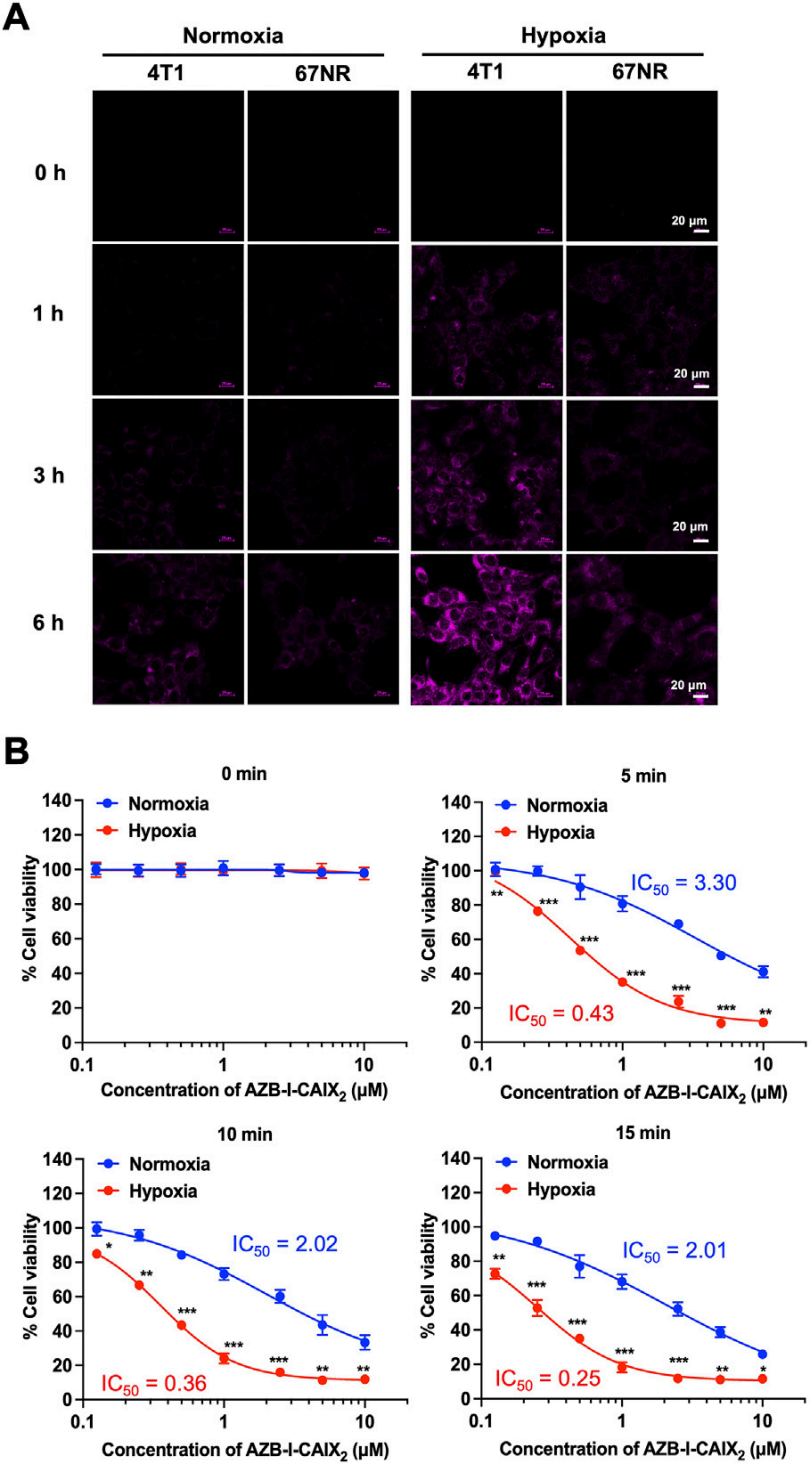
**(A)** Time-dependent cell internalization of AZB-I-CAIX_2_ under normoxia and hypoxia conditions of 4T1 and 67NR cells. **(B)** IC_50_ curves of 4T1 cells incubated with AZB-I-CAIX_2_ for 6 h before light illumination for 0 min, 5 min, 10 min, and 15 min.

4T1 and 67NR cells internalization experiments were performed under normoxia and hypoxia conditions. AZB-I-CAIX_2_ could be observed only from hypoxic 4T1 cells after 1 h incubation (Figure 7A) and the fluorescence increased in a time-dependent manner. After 6 h of treatment with AZB-I-CAIX_2_, 4T1 cells (normoxic and hypoxic) were exposed to light (660 nm) for various time periods (0 min, 5 min, 10 min, and 15 min). As shown in IC_50_ curves (Figure 7B), hypoxic cells were more sensitive to light compared to normoxic ones and the IC_50_ of hypoxic 4T1 cells was lower with longer exposure duration. These results suggest that under hypoxia, 4T1 expresses higher levels of CAIX, allowing AZB-I-CAIX_2_ to be internalized more efficiently, resulting in a greater accumulation of tumor cells. Importantly, PDT remained active even in the presence of low oxygen concentrations, indicating that the inclusion of a CAIX inhibitor can boost PDT’s therapeutic impact.

### 3.7 *In vivo a*cute toxicity study

**FIGURE 8:**
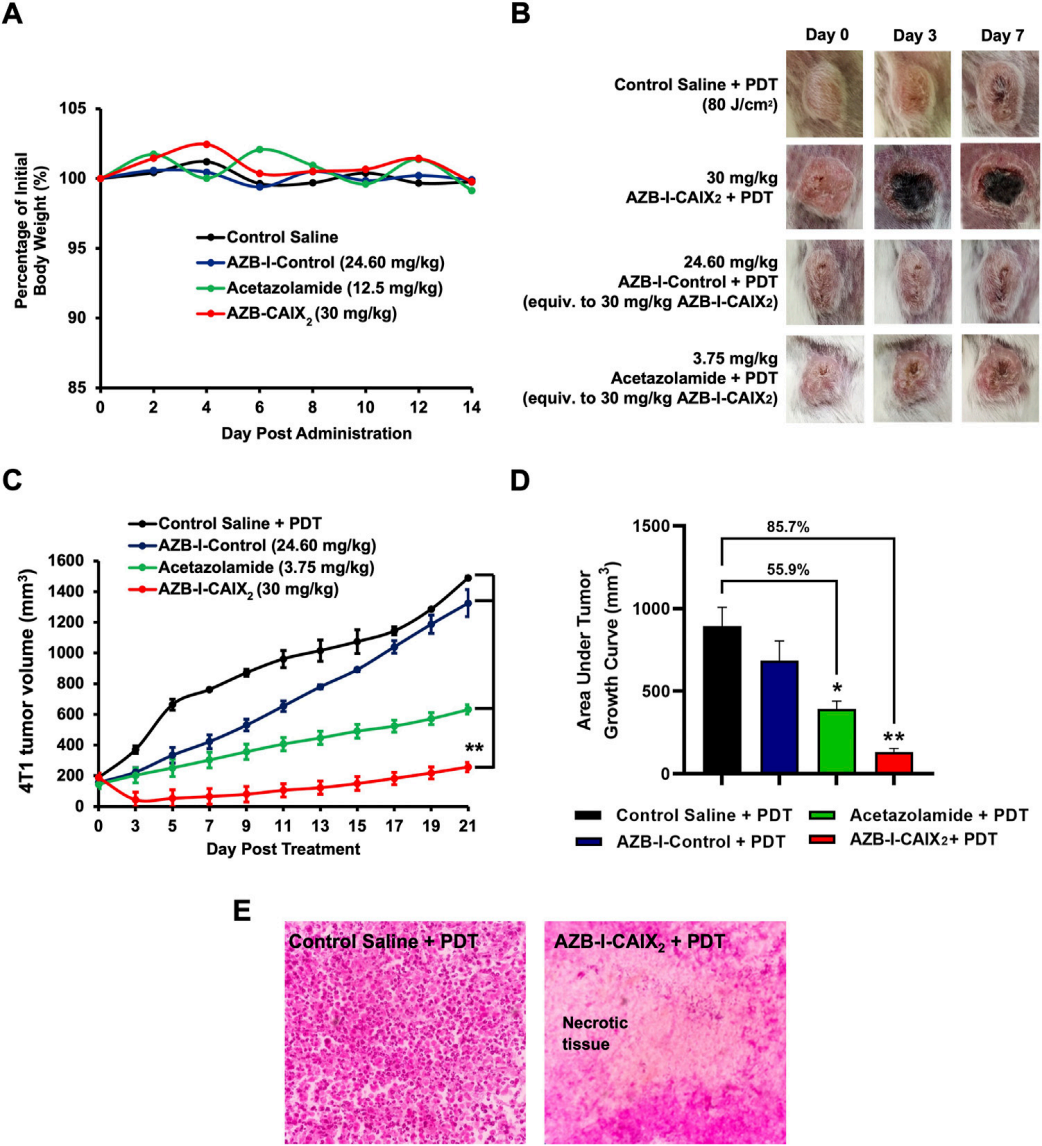
*In vivo* acute toxicity and antitumor efficacy of AZB-I-CAIX_2_, AZB-I-Control, and acetazolamide without irradiation. **(A)** Percentage of mice body weight (*n* = 2) for *in vivo* acute toxicity study throughout 14 days of observation. **(B)** Gross observation of tumor at days 0, 3, and 7 post-PDT. The figure in each group is representative of *n* = 6 mice. **(C)** 4T1 tumor volume post-AZB-I-CAIX_2_, AZB-I-Control and acetazolamide treatment, and PDT. ** *p <* 0.0001 for AZB-I-CAIX_2_ *vs* control saline, AZB-I-Control, and acetazolamide at all time points. **(D)** The area under the tumor volume in AZB-I-CAIX_2_, AZB-I-Control, and acetazolamide throughout 21 days of analysis. The graph represents mean ± SEM (*n* = 6), * *p* < 0.001 and ** *p* < 0.0001 compared to control saline, using One Way ANOVA (Dunnett’s). **(E)** H&E histological analysis of 4T1 tumor tissue post-three days of PDT. (Left) Control saline and PDT. (Right) 30 mg/kg AZB-I-CAIX2 and PDT with necrotic tissue. The picture shown is a representative of each group with similar results. Magnification: ×40.

The conjugates were then tested for acute toxicity in Balb/c mice. Intravenous administration of 30 mg/kg AZB-I-CAIX_2_, 24.60 mg/kg AZB-I-Control (equiv. to 30 mg/kg AZB-I-CAIX_2_), and 12.5 mg/kg acetazolamide (equiv. to 100 mg/kg AZB-I-CAIX_2_) showed no sign of toxicity such as apathy, weight loss, ruffled hair or behavioral changes up to 14 days of observation. No sudden weight loss was recorded, with constant weight (Gram) post-administration (Figure 8A). Histopathological analysis revealed no toxicity on the major organs such as heart, liver, spleen, lung, and kidney as shown in Supplementary Figure S14. Hence, 30 mg/kg of AZB-I-CAIX_2_ was chosen for the antitumor study.

### 3.8 *In vivo* photodynamic therapy

Antitumor efficacy of AZB-I-CAIX_2_, AZB-I-Control, and acetazolamide was compared in 4T1 tumor-bearing mice following PDT. In the absence of the photosensitizer, irradiation of the tumor tissue with light at an optimum light dose will not induce tumor necrosis. As shown in Figure 8B, tumor necrosis (black area) only appeared in the mice treated with AZB-I-CAIX_2_ at 30 mg/kg, starting at day-3 post-PDT. However, saline, AZB-I-Control, and acetazolamide control group-treated mice show progressive tumor growth, with no necrotic tumor tissue found from day-3 until day-7 post-PDT. Although the AZB-I-Control was used, tumor destruction was not observed, probably due to the poor accumulation in the tumor tissue. Hence, suggests the selective accumulation of AZB-I-CAIX_2_ in the 4T1 tumor tissue at 1 h post intravenous administration to mediate PDT-induced necrotic tissue. In comparing the tumor volume in every group as shown in Figure 8C, AZB-I-CAIX_2_ significantly reduced the tumor volume by 76.4% on day-3 compared to pre-PDT and remained smaller size throughout 21 days of observation (*p* < 0.0001). In contrast, administration of 24.60 mg/kg AZB-I-Control (Equiv. to 30 mg/kg AZB-I-CAIX_2_) and 3.75 mg/kg acetazolamide (Equiv. to 30 mg/kg AZB-I-CAIX_2_) treated groups showed no tumor shrinkage at day-3 post-PDT. An analysis in comparing entire tumor volume over time was performed between groups. As shown in Figure 8D, the AZB-I-CAIX_2_ treatment group has 85.7% lower tumor volume (*p* < 0.0001), followed by 55.9% in acetazolamide (*p <* 0.001) and 24.4% in AZB-I-Control (*p =* 0.232) compared to the control group. In order to confirm the tumor undergo necrosis post-PDT, the tumor tissue in AZB-I-CAIX_2_ treated mice post 3 days of PDT were harvested for H&E histological analysis. PDT necrosis was mostly confluent in tumor tissue treated with AZB-I-CAIX_2_ compared to control saline treated group post PDT, with many viable tumor cells (Figure 8E). This suggested that the CAIX_2_ ligand has improved the delivery of AZB-I to CAIX+ tumor. Administration of acetazolamide showed 55.9% lower tumor volume compared to the control group, this is parallel with other studies as reported CAIX-Inhibitor acetazolamide able to reduce the tumor growth, invasiveness, and proliferation of cancer cells (Li et al., 2007; Cianchi et al., 2010).

## 4 Conclusion

The combination of CAIX inhibition and NIR photosensitizer has tremendous potential for overcoming hypoxic restrictions in PDT. We designed a CAIX-targeting NIR photosensitizer (AZB-I-CAIX_2_) that contains two acetazolamide and aza-BODIPY moieties to offer a singlet oxygen-producing photosensitizer while permitting fluorescence-based cell internalization tracking. AZB-I-control was a control, a comparable aza-BODIPY structure without acetazolamide units, that only has a PDT impact and no ability to target. *In vitro* studies revealed that AZB-I-CAIX_2_ was preferentially localized to the ER and selectively internalized in human breast cancer cells (MDA-MB-231) that overexpress CAIX (CAIX+), whereas AZB-I-control did not show any cell uptake. The selectivity of AZB-I-CAIX_2_ to CAIX was also confirmed by inhibitory effect and co-cultured of CAIX+ and CAIX- cells. AZB-I-CAIX_2_ also produced singlet oxygen quickly after being exposed to 660 nm light and showed selective photo-induced cell cytotoxicity at low doses. Furthermore, under hypoxia-induced CAIX expression in 4T1 cells, AZB-I-CAIX_2_ revealed a robust fluorescence response and demonstrated efficient cancer cell ablation by PDT. Finally, *in vivo* experiments in mice revealed that AZB-I-CAIX_2_ had no acute toxicity when used without irradiation, while dramatically lowering tumor mass following light exposure when compared to mice treated with AZB-I-control and acetazolamide. As a result, AZB-I-CAIX_2_ could be a useful targeting agent for CAIX-expressing cells, with increased therapeutic efficacy in alleviating PDT-induced hypoxia due to CAIX suppression.

## Data Availability

The original contributions presented in the study are included in the article/Supplementary Material; further inquiries can be directed to the corresponding author.
