# Supplementary material for: Aza-BODIPY based carbonic anhydrase IX: Strategy to overcome hypoxia limitation in photodynamic therapy
Source: Front Chem. 2022 Nov 2;10:1015883. doi: 10.3389/fchem.2022.1015883 (PMC9666899; doi:10.3389/fchem.2022.1015883)
Supplement: Supplementary file 1 [file DataSheet1.PDF]

Aza-BODIPY Based Carbonic Anhydrase IX: Strategy to Overcome Hypoxia Limitation in Photodynamic Therapy

Supporting Information

1. General Procedures……………………………………………………………………..2

2. ^{1}H, ^{13}C, ^{19}F, and 2D NMR and Mass spectra………….....…………………….……….……….3

3. HPLC analysis of **AZB-I-CAIX$_2$** and **AZB-I-Control**……………………………………......17

3. UV/Vis and fluorescence spectroscopic data of **AZB-I-Control** ………………………...…..18

5. Singlet Oxygen Quantum Yields……………………………………………………..…….19

6. Stability of **AZB-I-CAIX$_2$** and **AZB-I-Control** in FBS…………………………………….…21

7. Biological studies experiments…………………………...………………………..…….22

1. General Procedures

In all reactions, glassware was oven-dried before use. All the reagents have been purchased from commercial sources (Sigma-Aldrich, TCI, Carlo Erba, ACROS, and Merck) and used without further purification. Column chromatography purifications were performed using a stationary phase using silica gel (Merck). Analytical thin-layer chromatography (TLC) was performed on TLC Silica gel 60 F254 (Merck) and visualized with a UV cabinet (254 and 365 nm).

^{1}H, ^{13}C, ^{19}F, COSY, and HSQC NMR spectra were recorded on a Bruker-500 MHz spectrometer at room temperature. Chemical shifts of ^{1}H NMR spectra were reported in ppm and calibrated from the residue solvent peak, $CDCl_3$ (7.24 ppm), MeOD-d_4 (3.31 ppm), and DMSO-d_6 (2.50 ppm). ^{1}H NMR data are reported as follows: chemical shift, multiplicity (s = singlet, d = doublet, t = triplet, m = multiplet, dd =doublet of doublet), coupling constants, and a number of protons. ^{13}C NMR spectra were also recorded in ppm from the solvent resonance, $CDCl_3$ (77.23 ppm), MeOD-d_4 (49.00 ppm), and DMSO-d_6 (39.50 ppm). Mass spectra (MS) were measured under high-resolution ESI^{+} conditions.

2. ^{1}H and ^{13}C NMR and Mass spectra

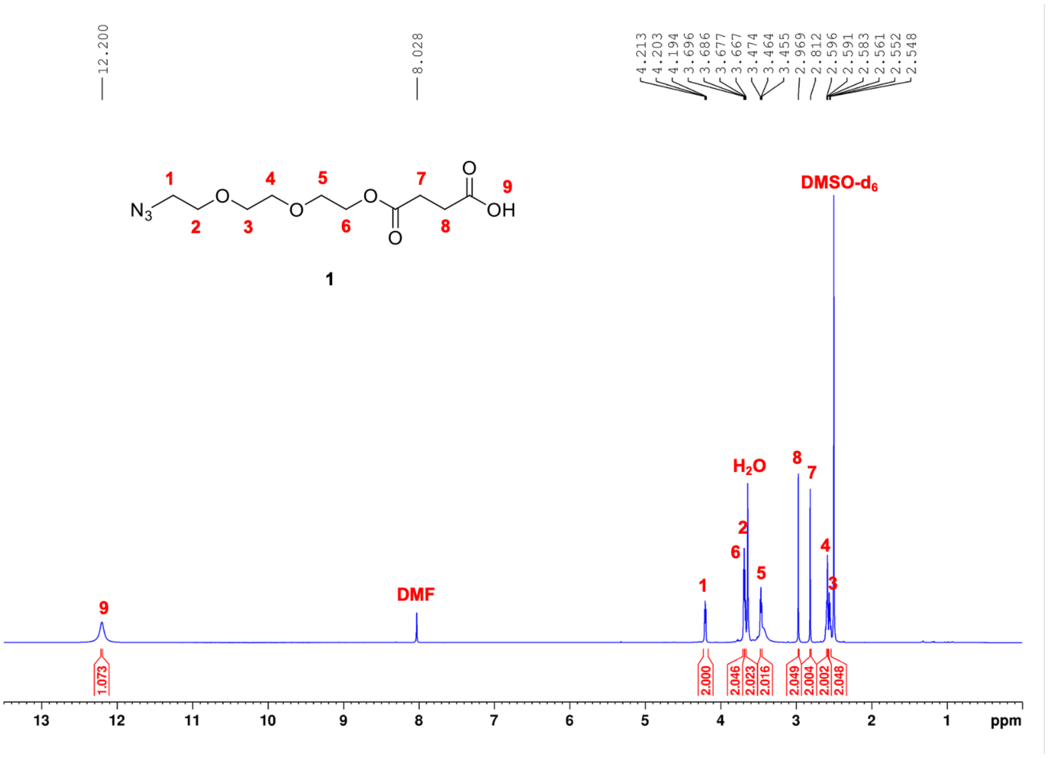

^{1}H NMR of compound **1** in DMSO-d6

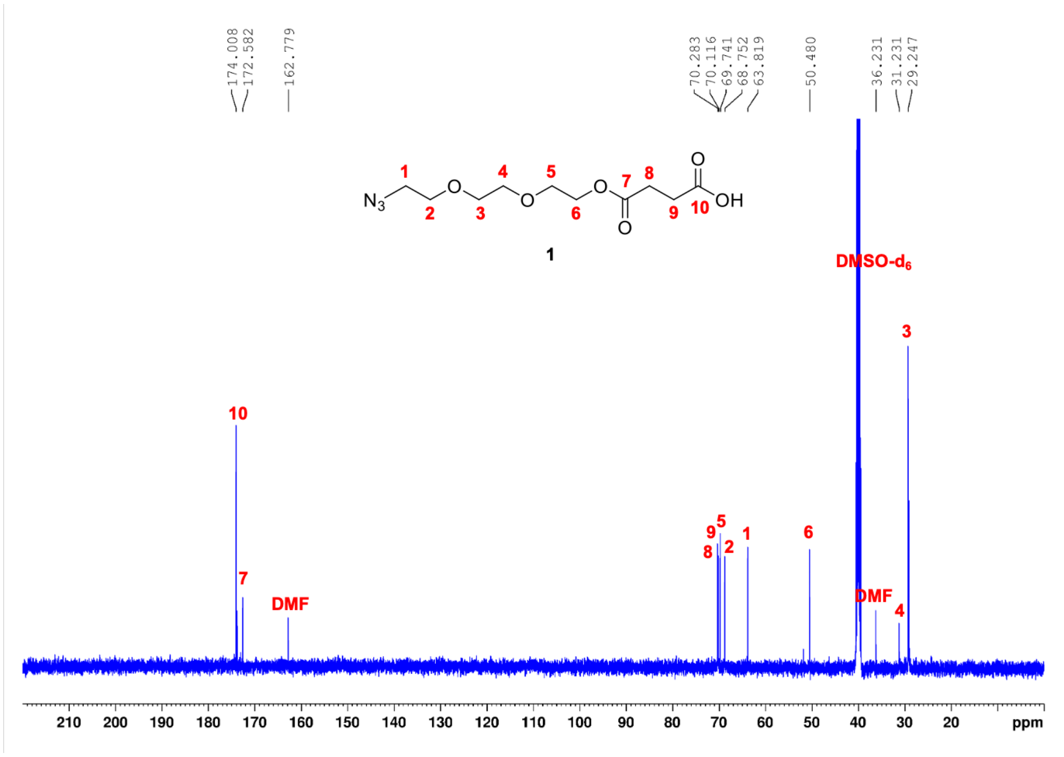

^{13}C NMR of compound **1** in DMSO-d6

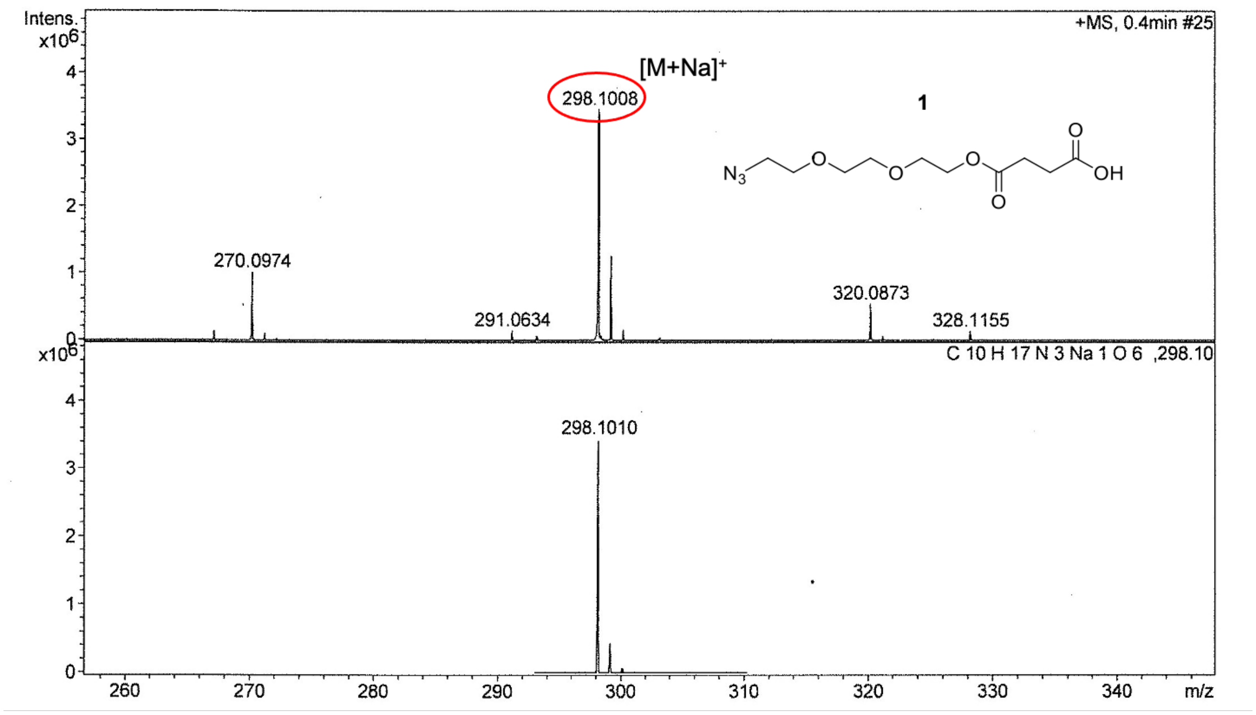

High-resolution ESI $^+$ MS of compound **1**

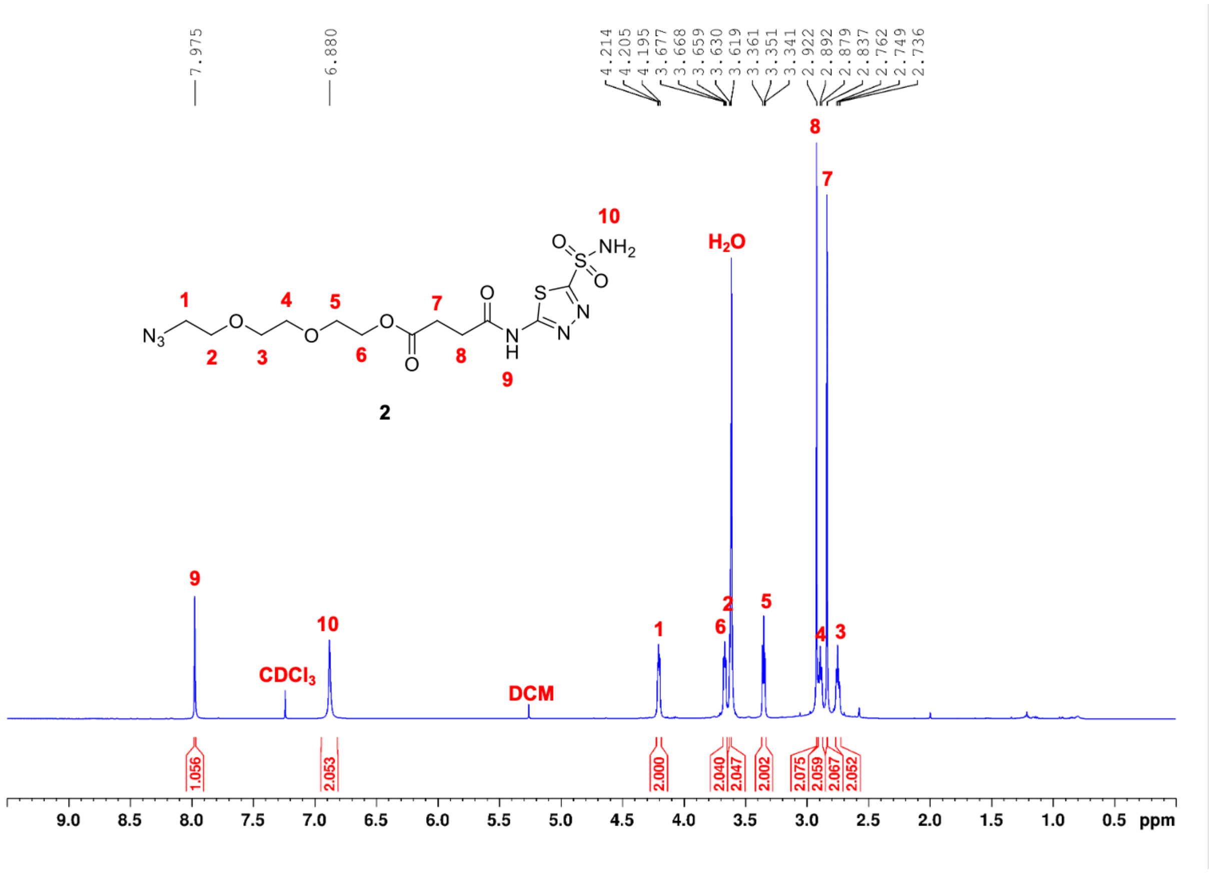

^{1}H NMR of compound **2** in $CDCl_3$

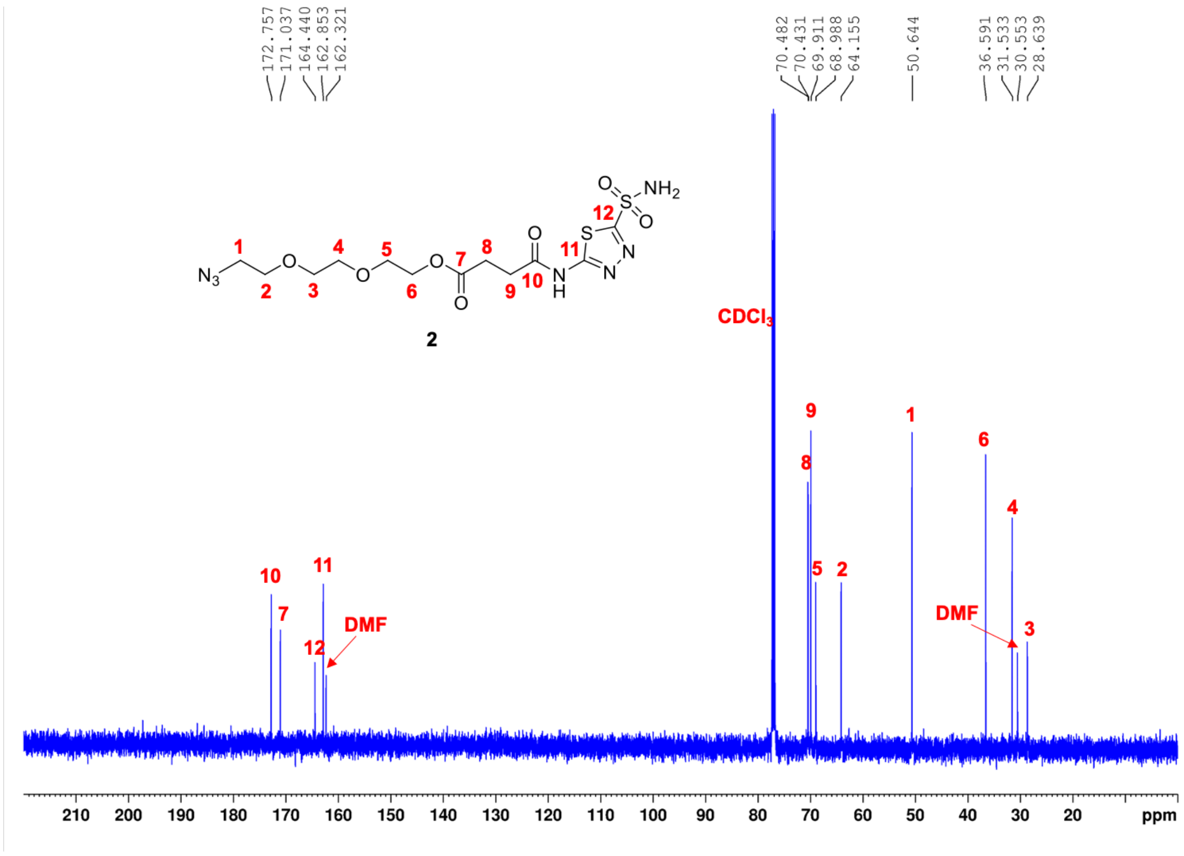

^{13}C NMR of compound **2** in $CDCl_3$

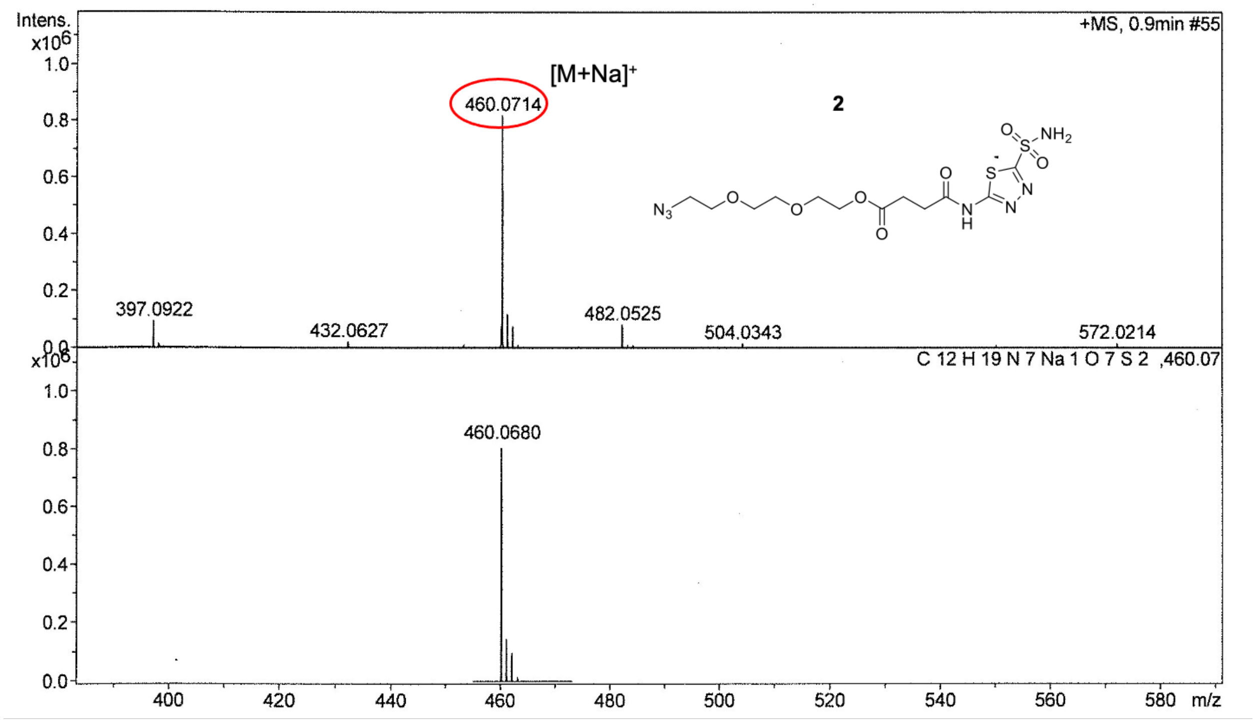

High-resolution ESI $^+$ MS of compound **2**

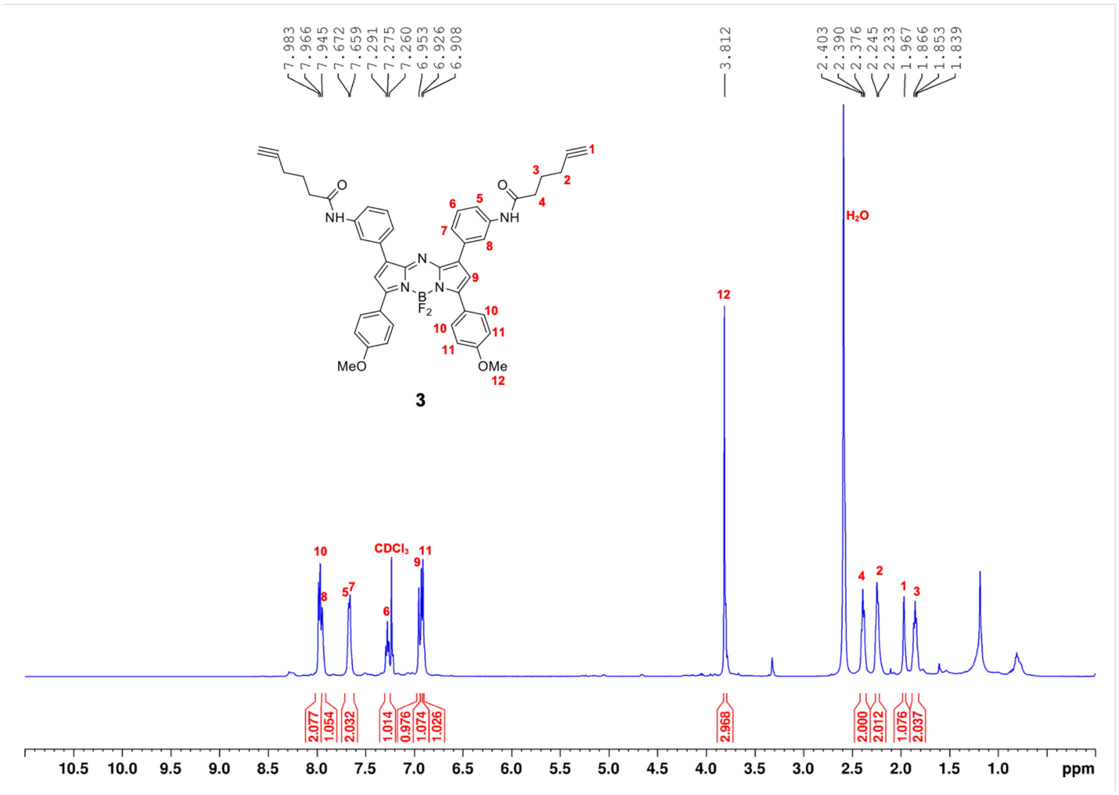

^{1}H NMR of compound **3** in $CDCl_3$

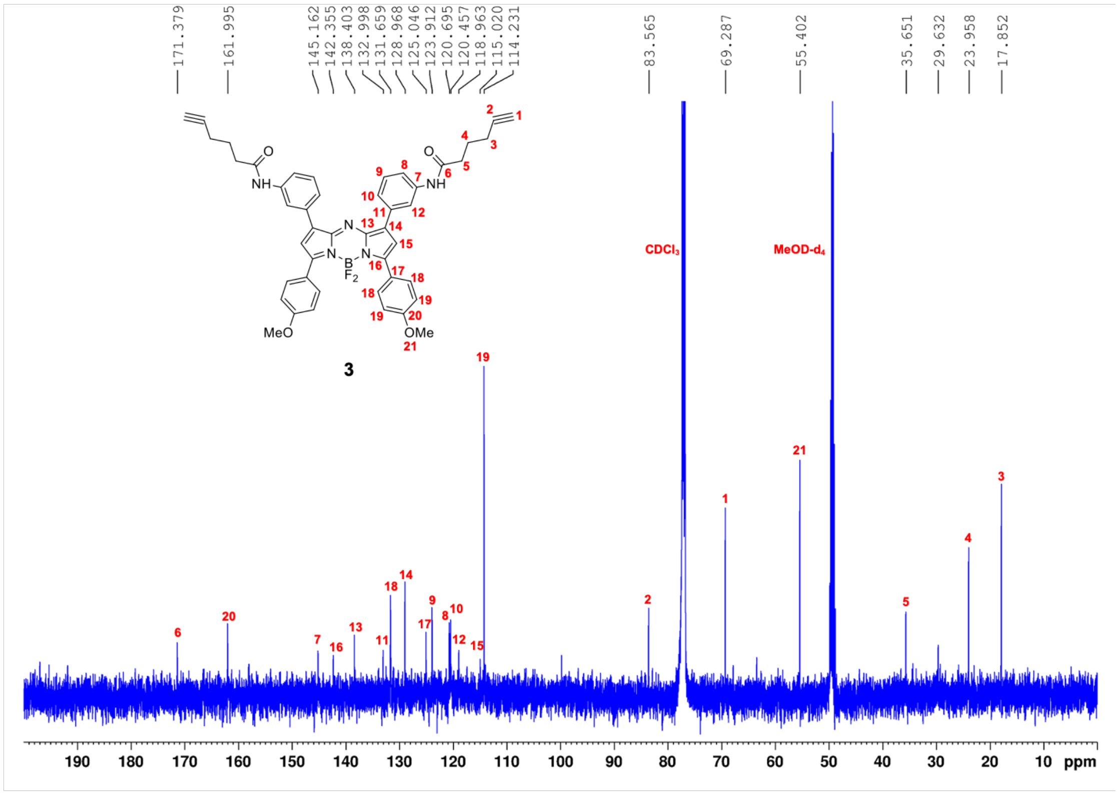

^{13}C NMR of compound **3** in $CDCl_3$

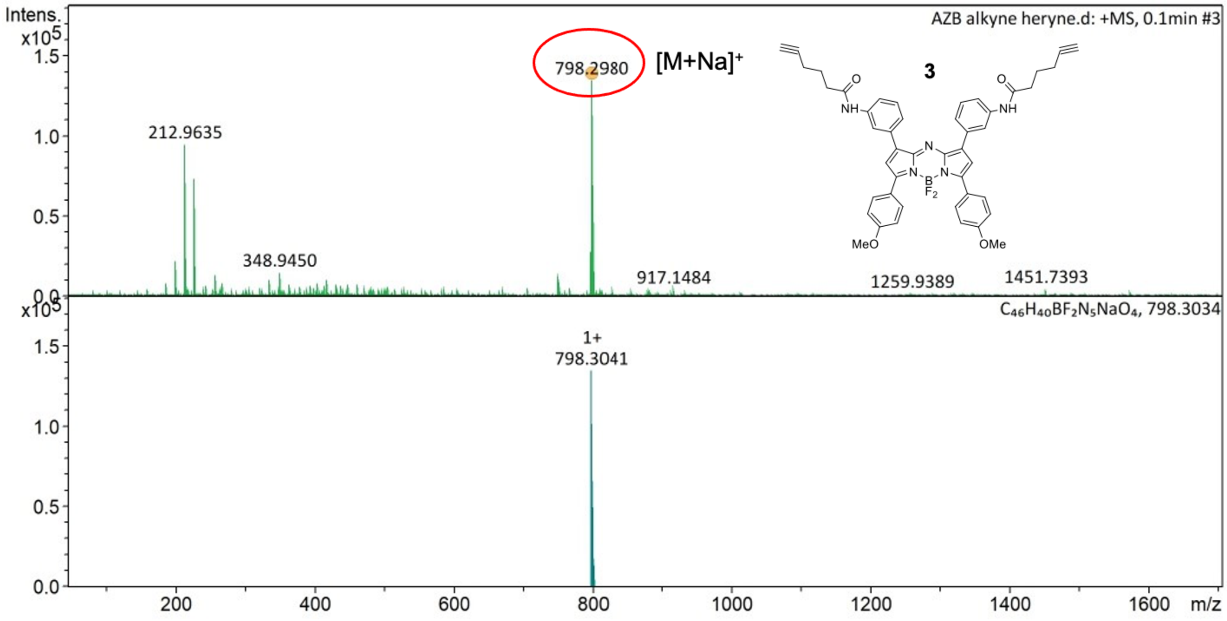

High-resolution ESI $^+$ MS of compound **3**

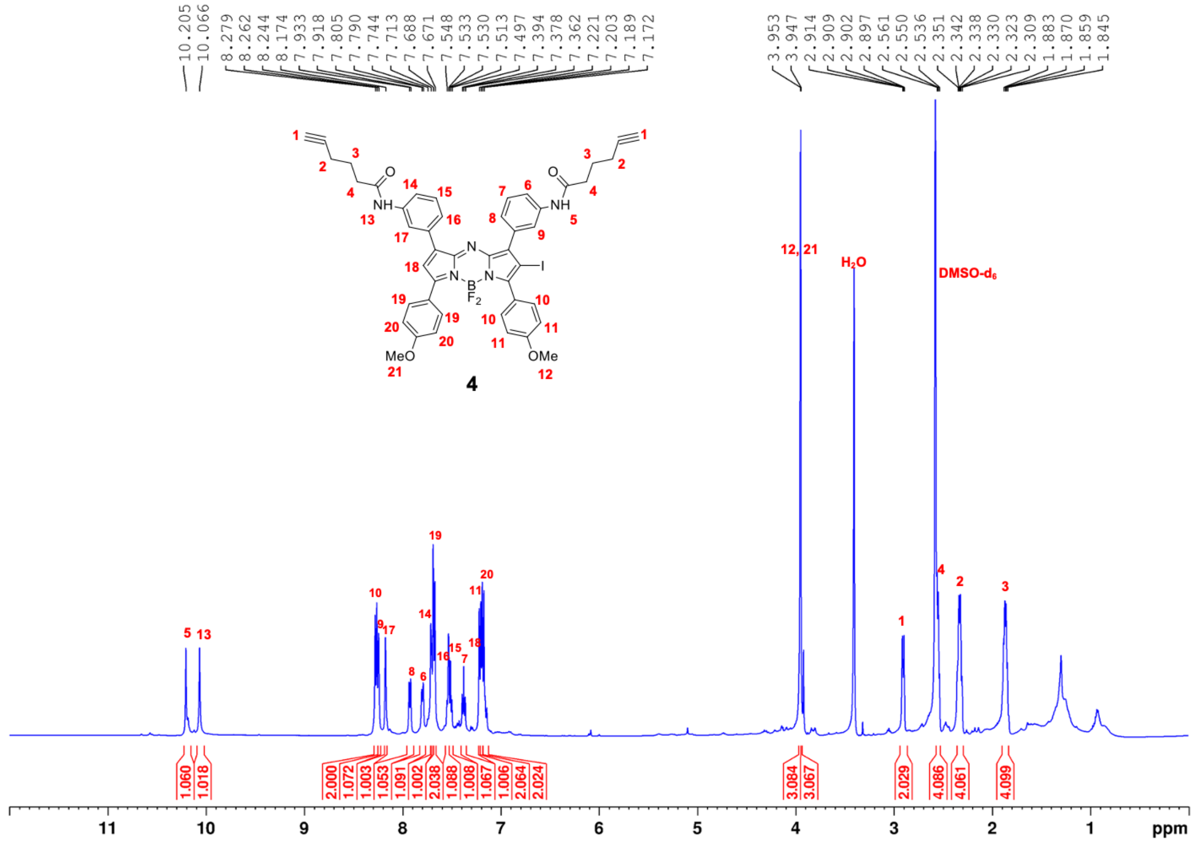

^{1}H NMR of compound **4** in DMSO-d$_6$

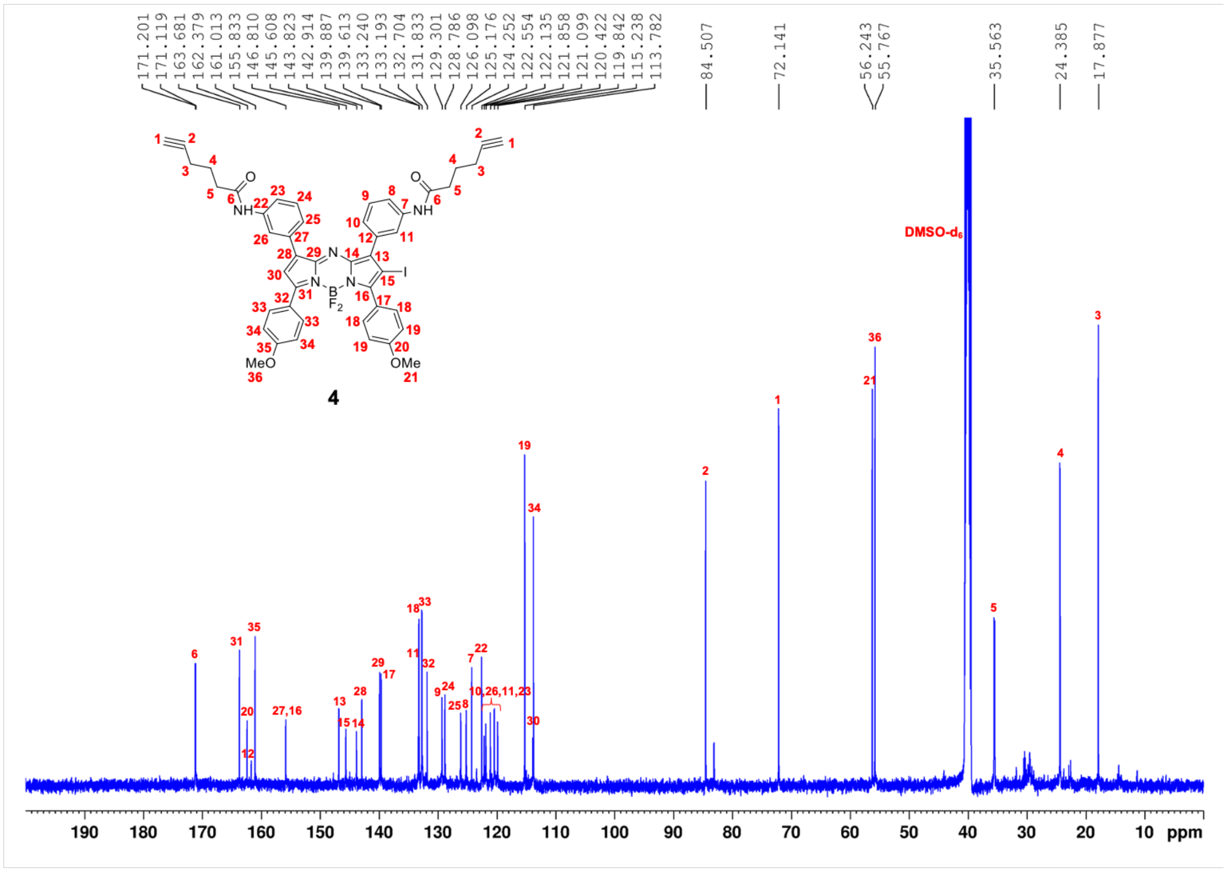

^{13}C NMR of compound **4** in DMSO-d$_6$

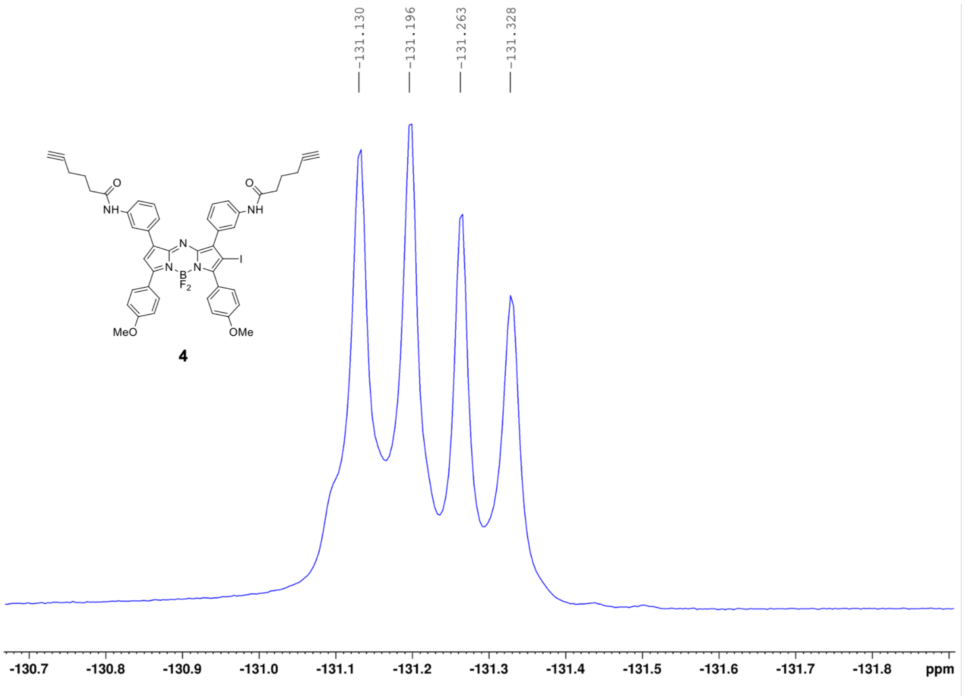

^{19}F NMR of compound **4** in DMSO-d$_6$

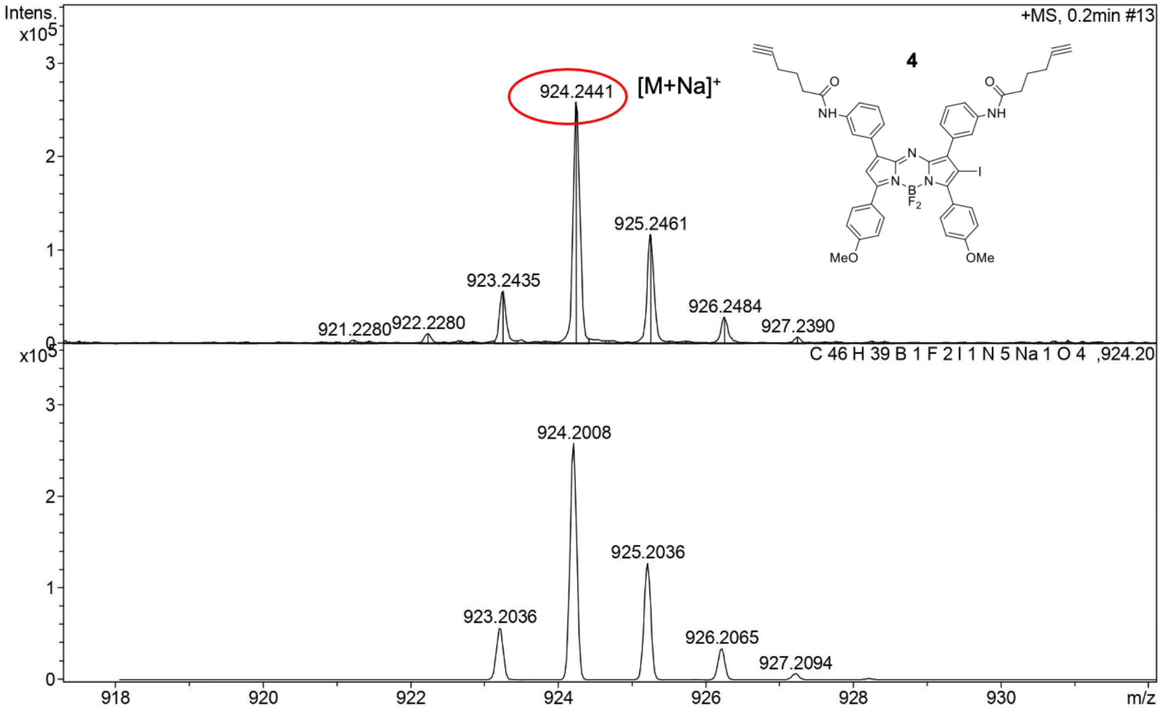

High-resolution ESI $^+$ MS of compound **4**

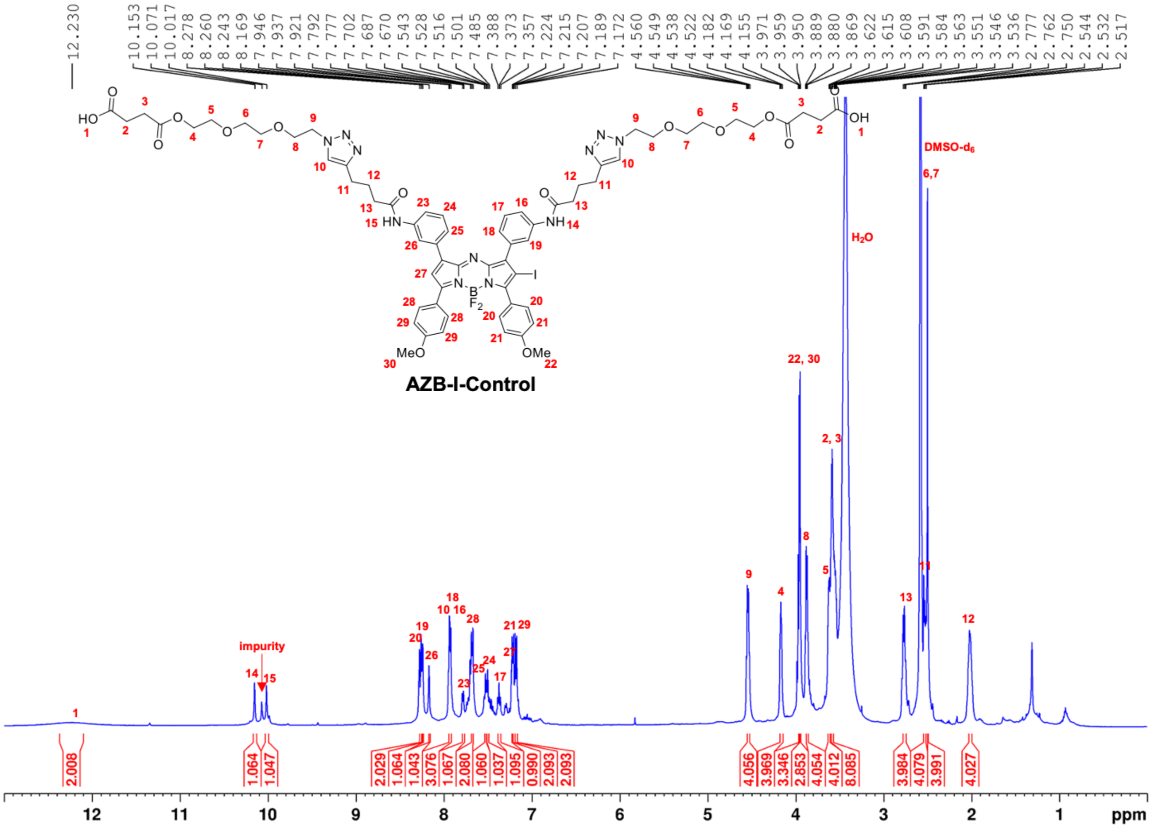

^{1}H NMR of compound **AZB-I-Control** in DMSO-d_6

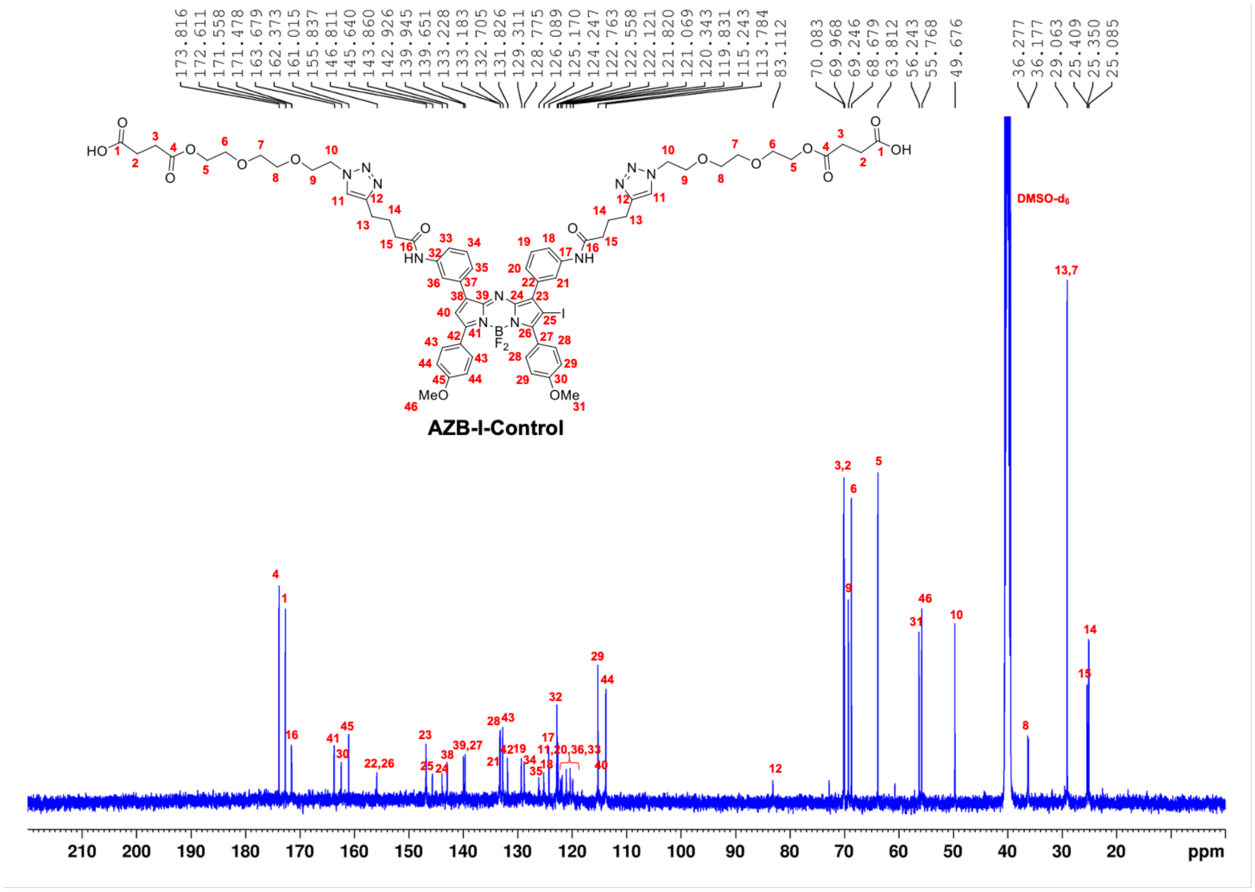

^{13}C NMR of compound **AZB-I-Control** in DMSO-d$_6$

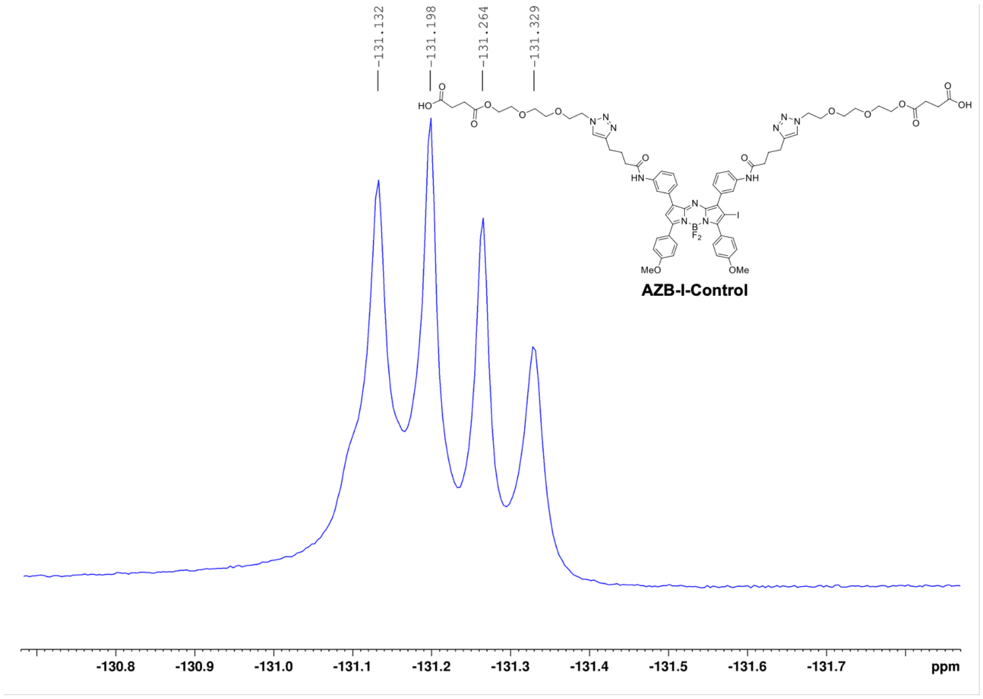

^{19}F NMR of compound **AZB-I-Control** in DMSO-d$_6$

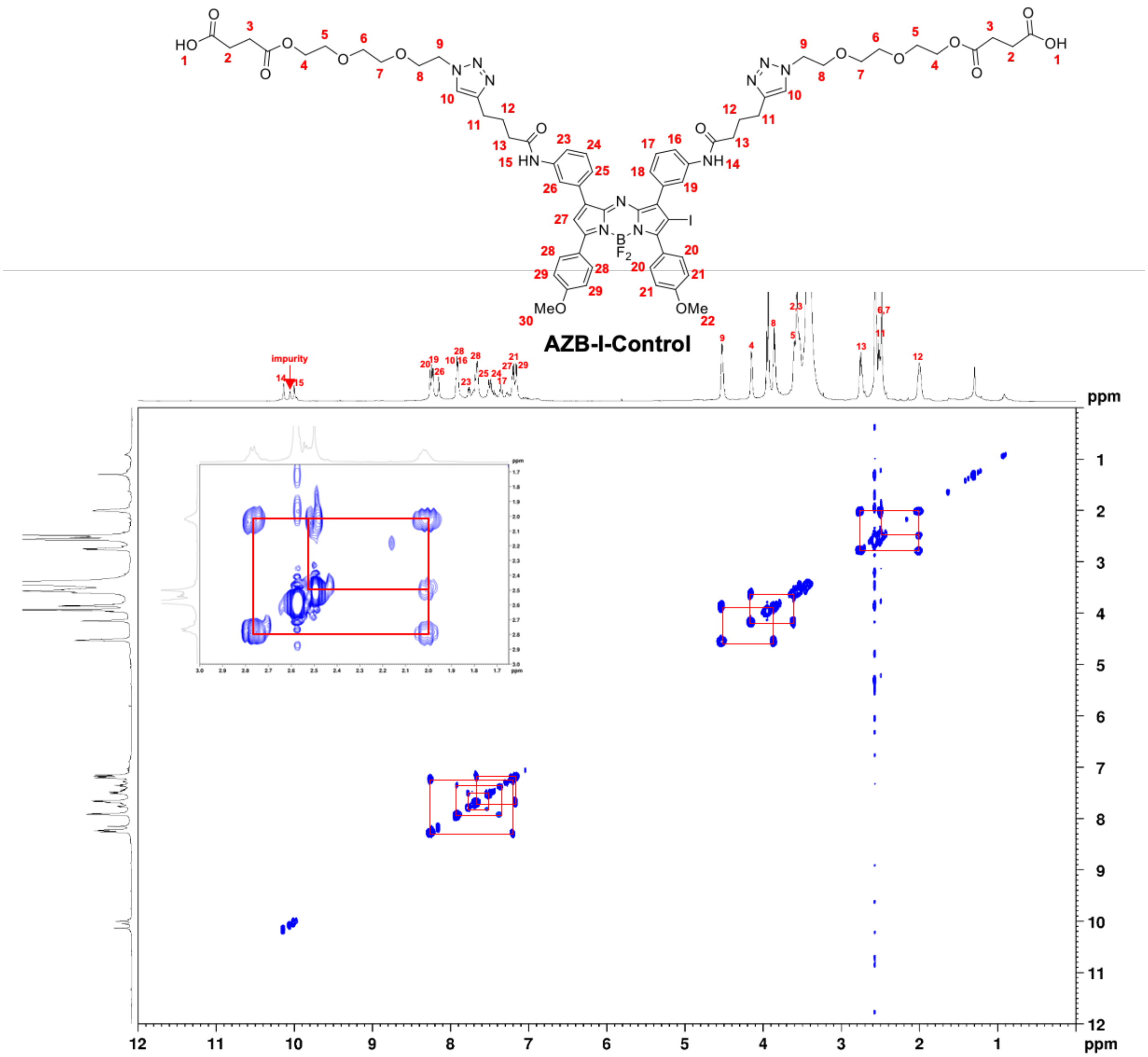

COSY NMR of compound **AZB-I-Control** in DMSO-d_6

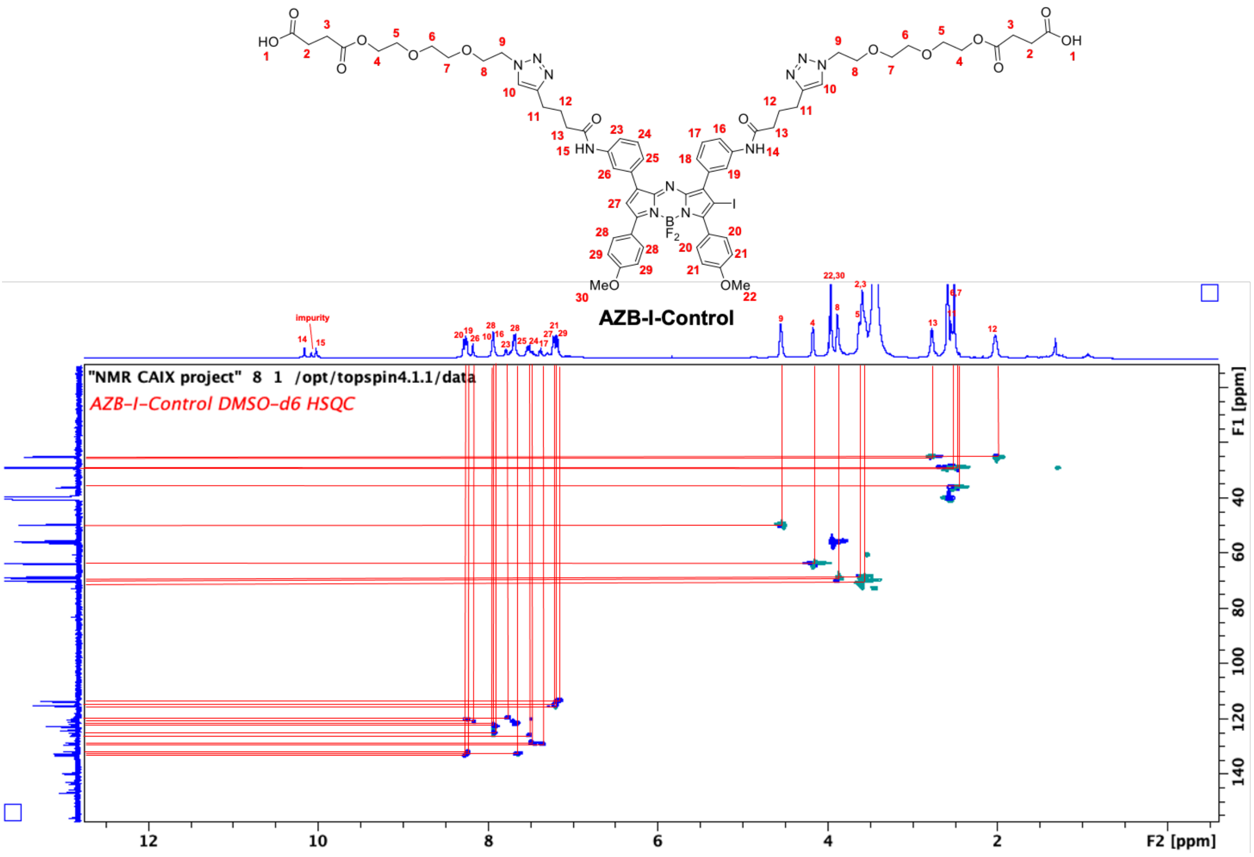

HSQC NMR of compound **AZB-I-Control** in DMSO-d_6

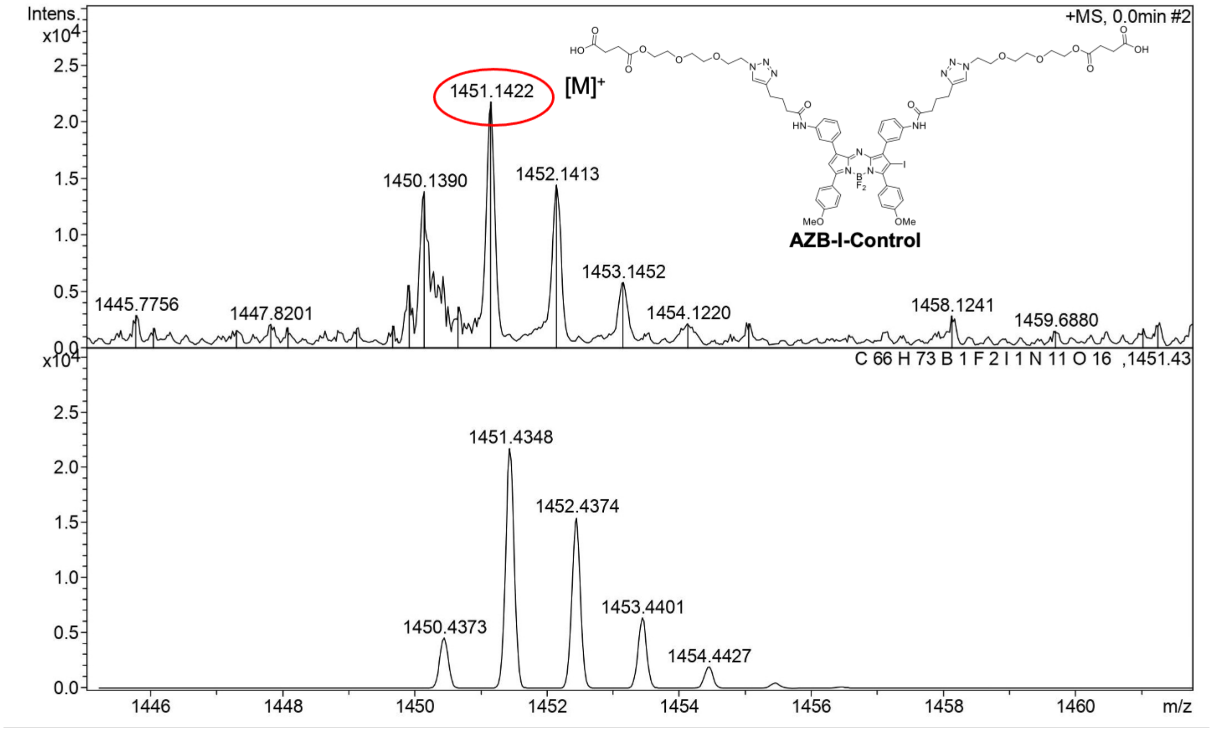

High-resolution ESI $^+$ MS of compound **AZB-Control-I**

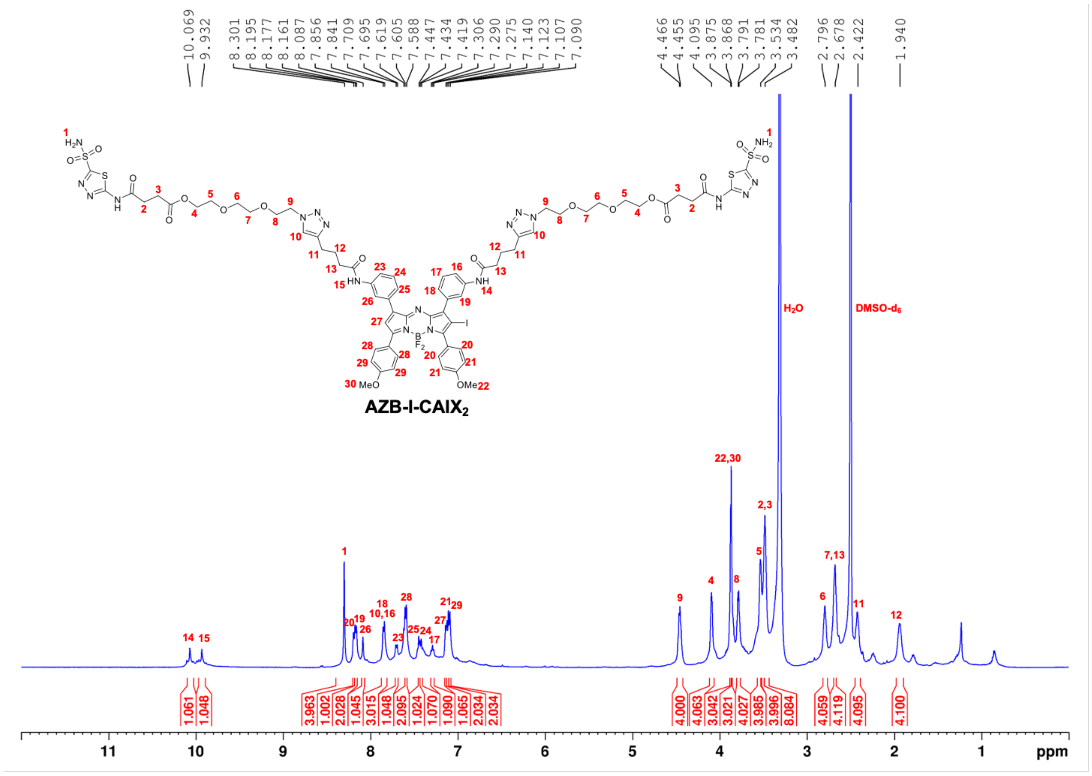

^{1}H NMR of compound **AZB-I-CAIX$_2$** in DMSO-d$_6$

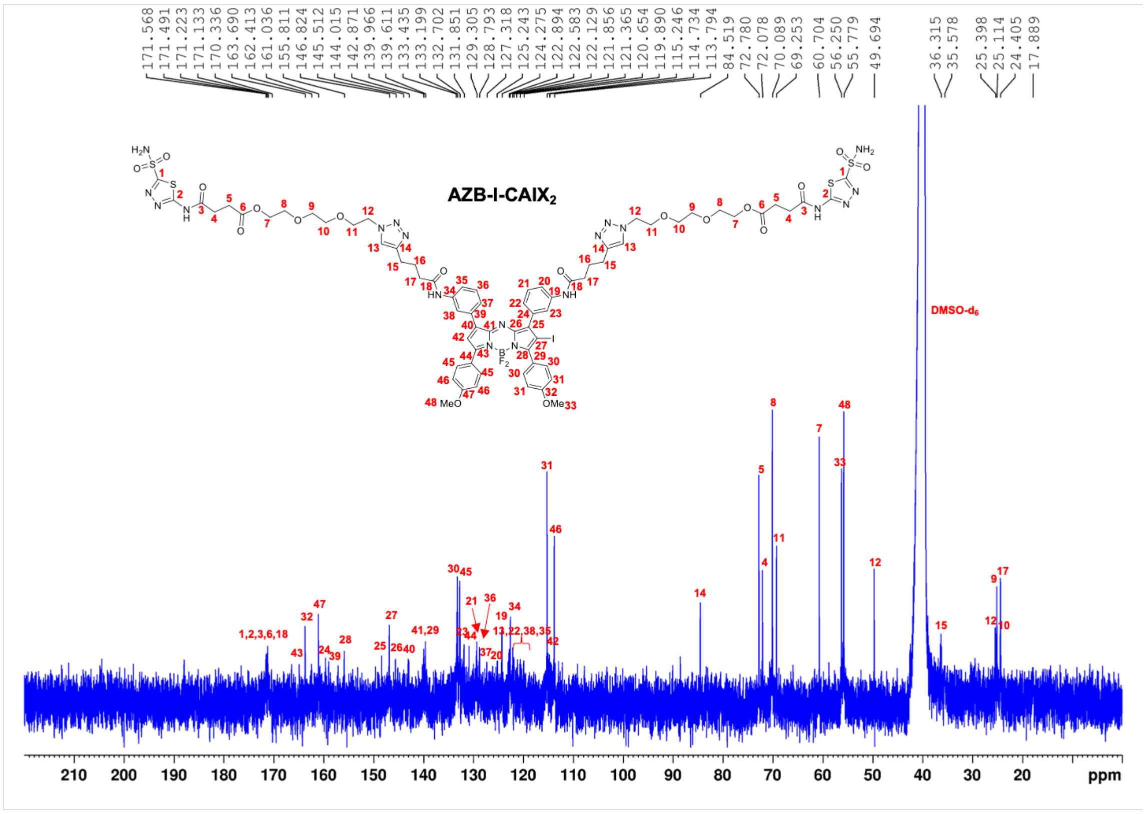

^{13}C NMR of compound **AZB-I-CAIX$_2$** in DMSO-d$_6$

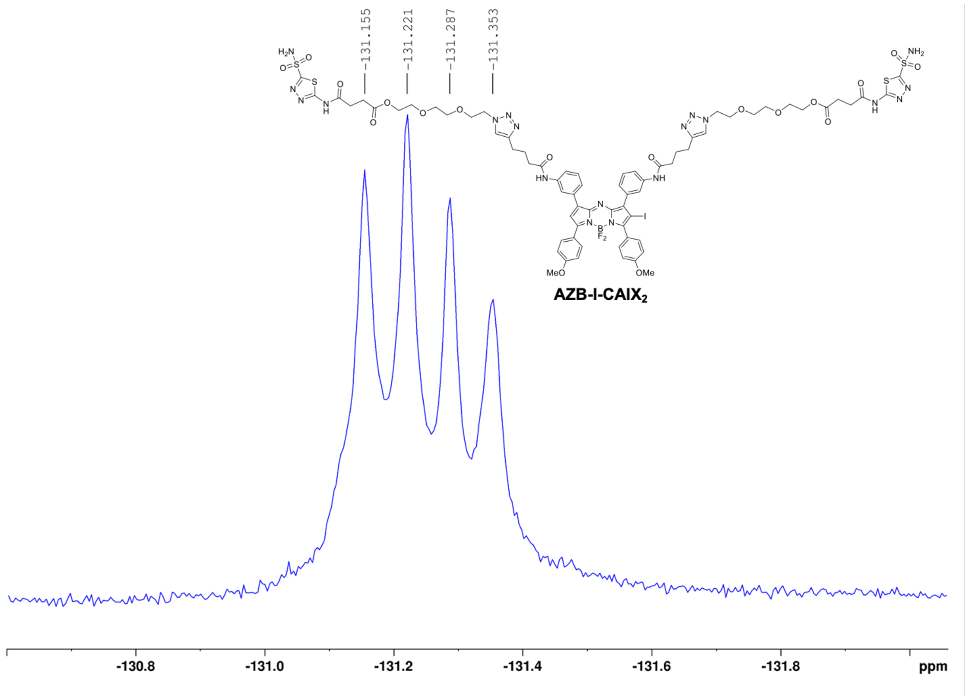

^{19}F NMR of compound **AZB-I-CAIX$_2$** in DMSO-d$_6$

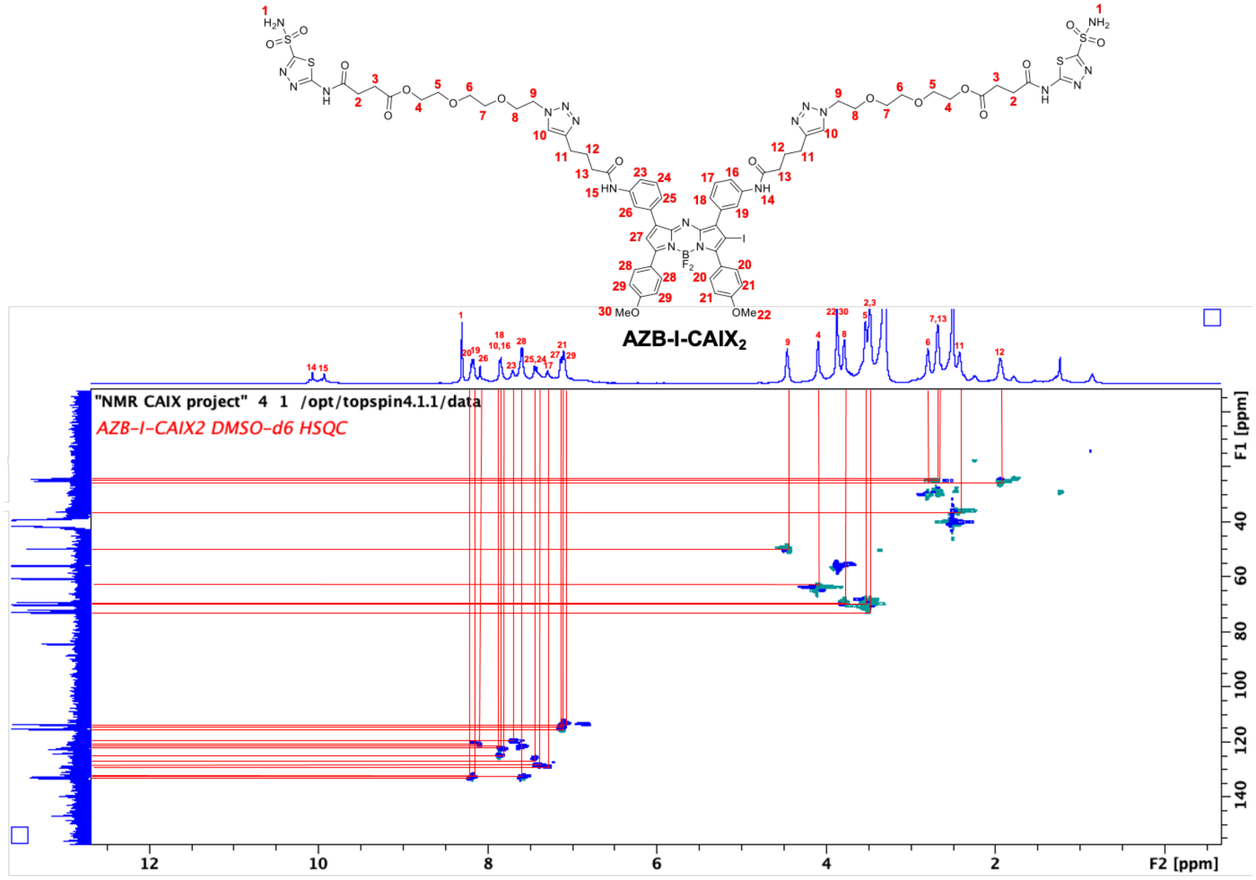

HSQC NMR of compound **AZB-I-CAIX$_2$** in DMSO-d$_6$

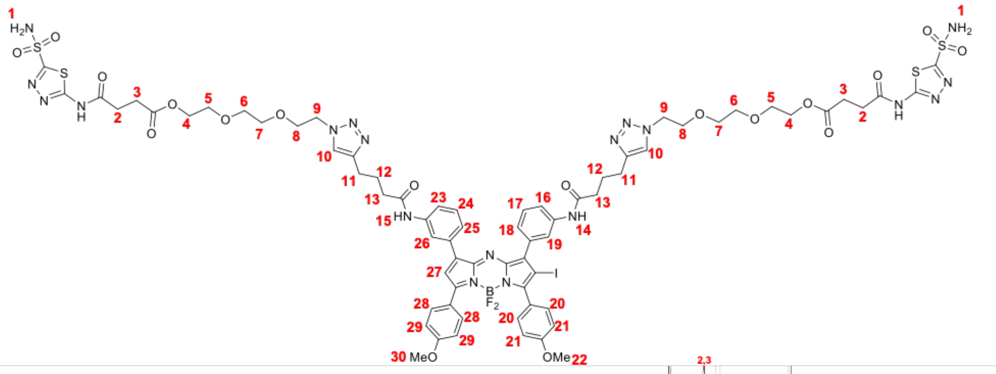

AZB-I-CAIX₂

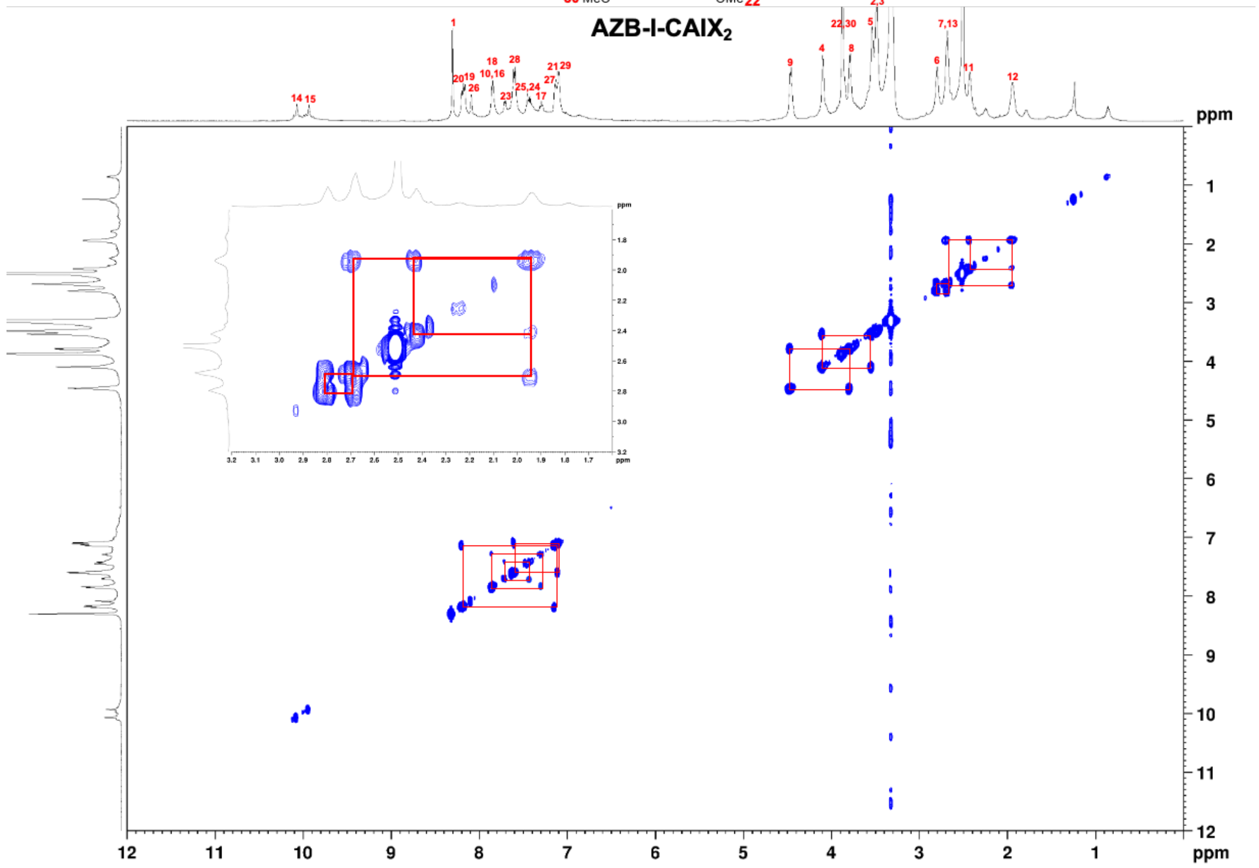

COSY NMR of compound **AZB-I-CAIX$_2$** in DMSO-d$_6$

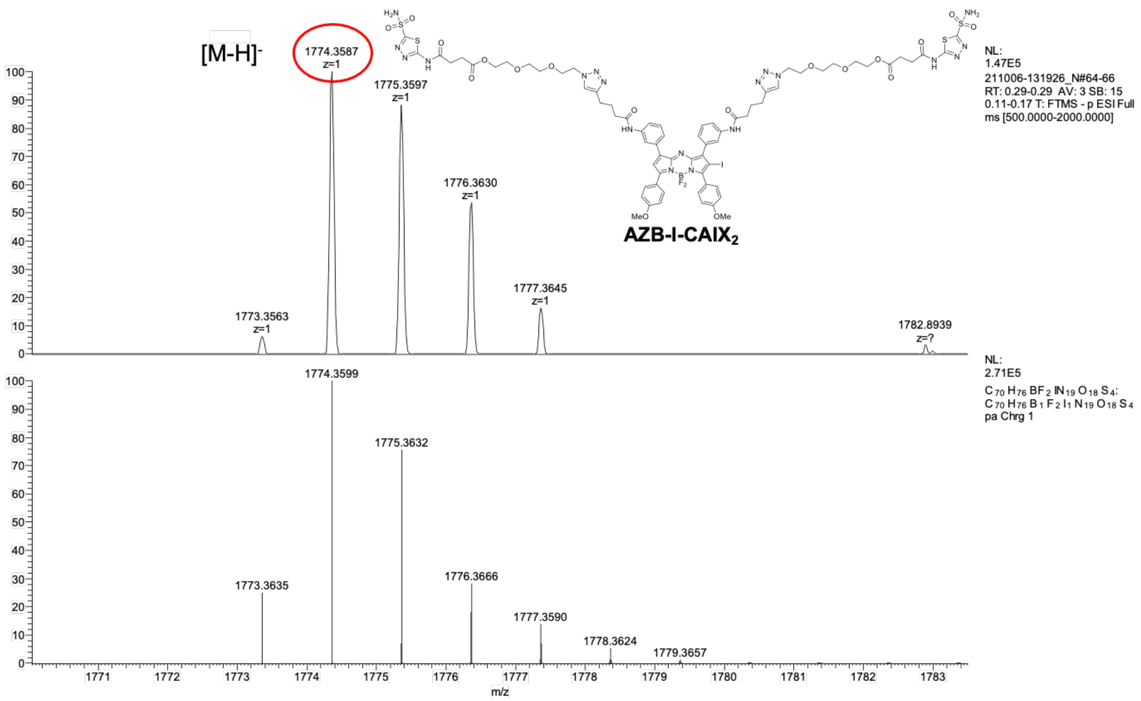

High-resolution ESI $^{-}$ MS of compound **AZB-I-CAIX$_2$**

3. HPLC analysis of AZB-I-CAIX$_2$ and AZB-I-Control

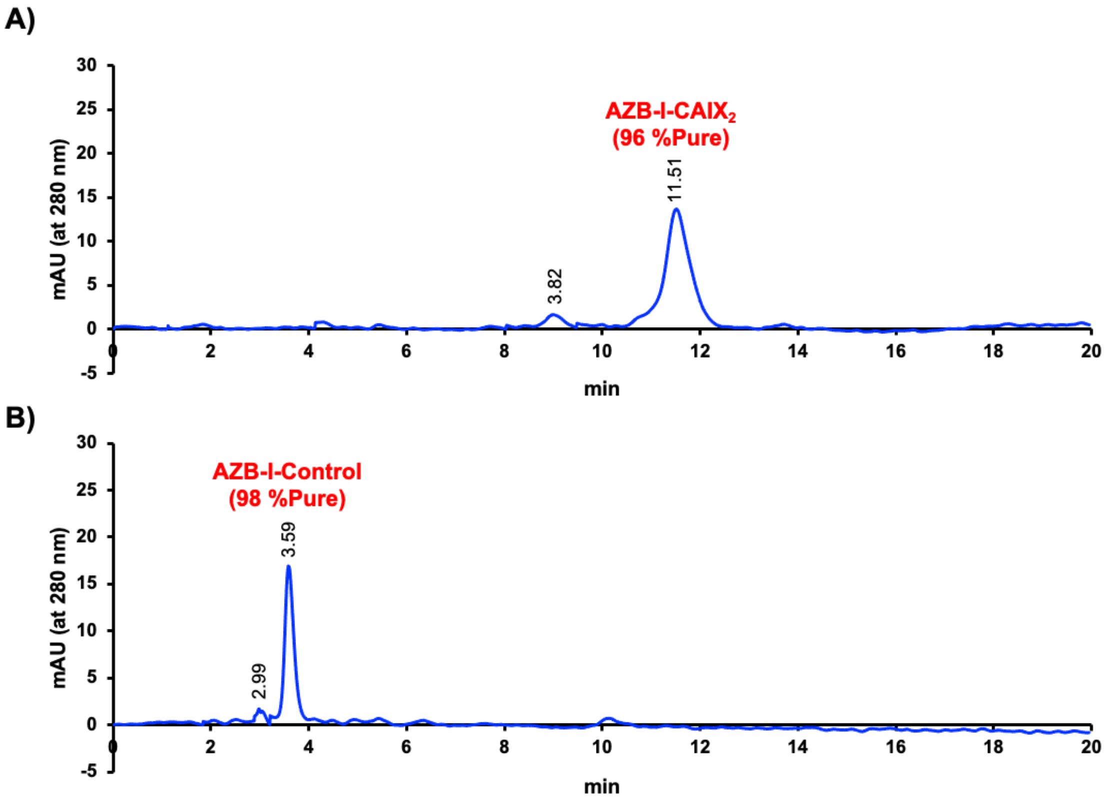

Fig S1. HPLC analysis of A) **AZB-I-CAIX$_2$** and B) **AZB-I-Control** at 280 nm detection. Reverse-phase HPLC was performed on an Agilent HPLC 1100 and ZORBAX Eclipse XDB-C18 (4.6 mm × 150 mm, 5 µm ID) column using a mobile phase of solvent A = 20 mM ammonium acetate, solvent B = 100 % acetonitrile, isocratic 50 % A: B, and flow rate 1 mL/min. The analysis was monitored by a UV-Vis detector at a wavelength of 280 nm.

4. UV/Vis and fluorescence spectroscopic data of AZB-I-Control

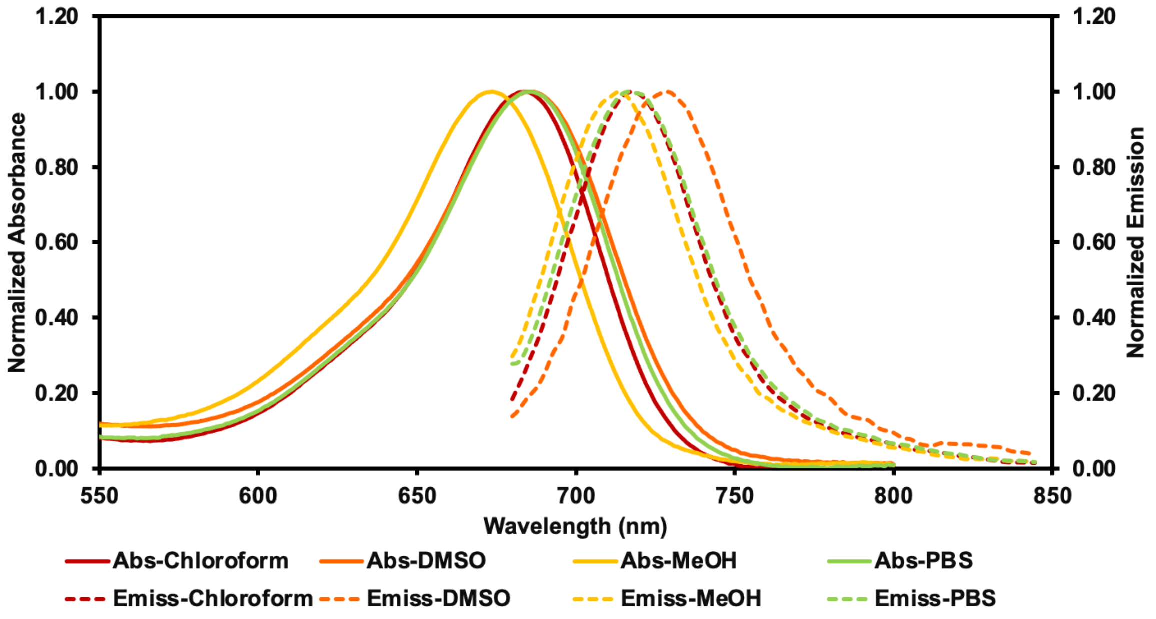

Fig S2. UV-Vis and fluorescent spectra of **AZB-I-Control** in different solvents

5. Singlet Oxygen Quantum Yields

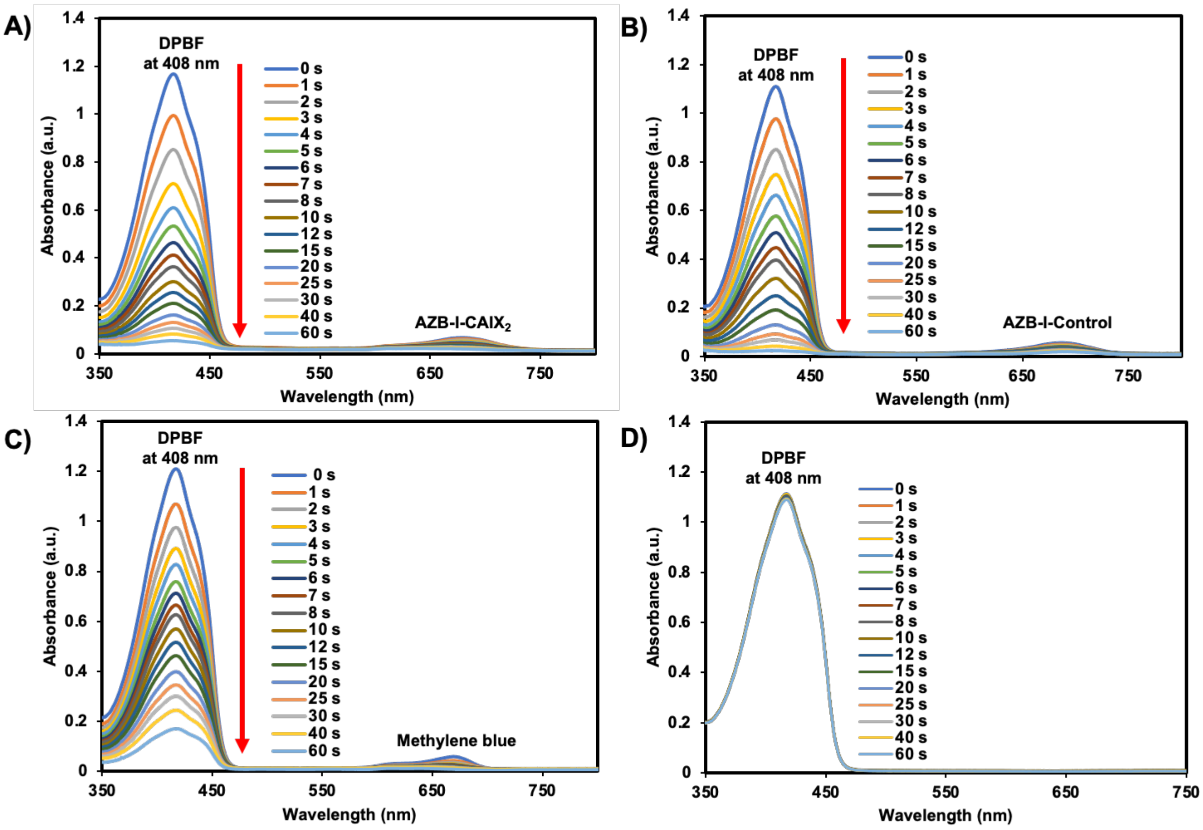

Fig S3. Absorbance response of DPBF at 408 nm upon irradiation after exposure with the lamp (8.7 mW cm^{-2}) during 0-60 s in DMSO. A) **AZB-I-CAIX$_2$**, B) **AZB-I-Control**, C) methylene blue, and d) DMSO only.

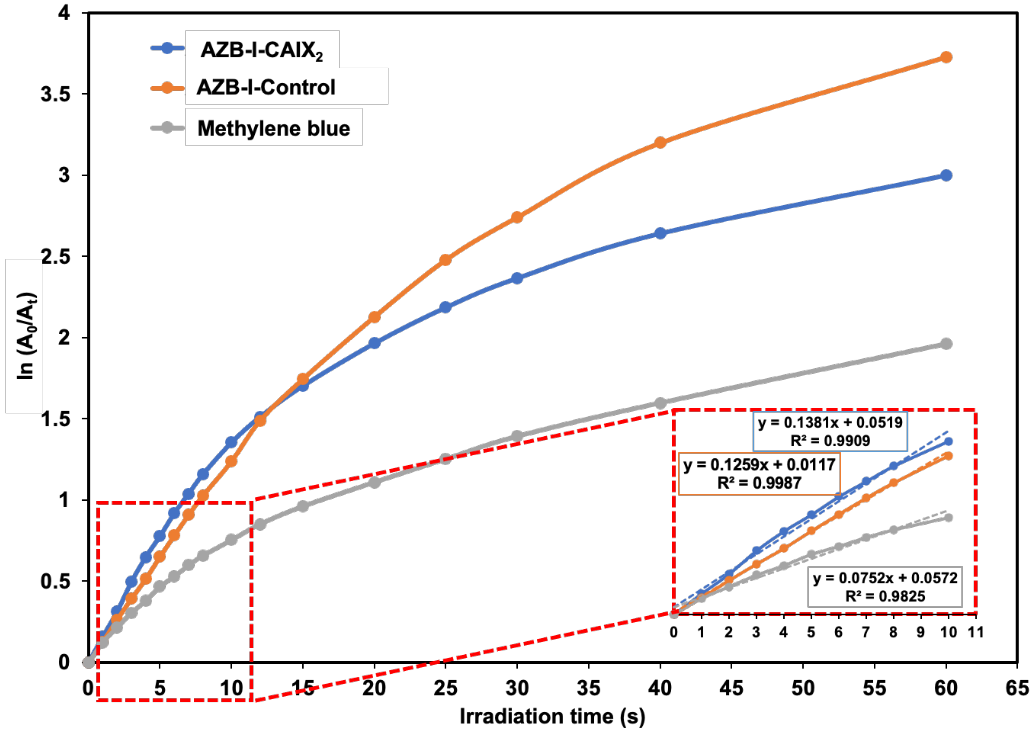

Fig S4. Reduction of DPBF absorbance response at 408 nm upon irradiation time in DMSO by **AZB-I-CAIX$_2$**, **AZB-I-Control,** and methylene blue and linear plots in initial rate (0 - 10 s) of DPBF absorbance response at 408 nm.

6. Stability of AZB-I-CAIX$_2$ and AZB-I-Control in FBS

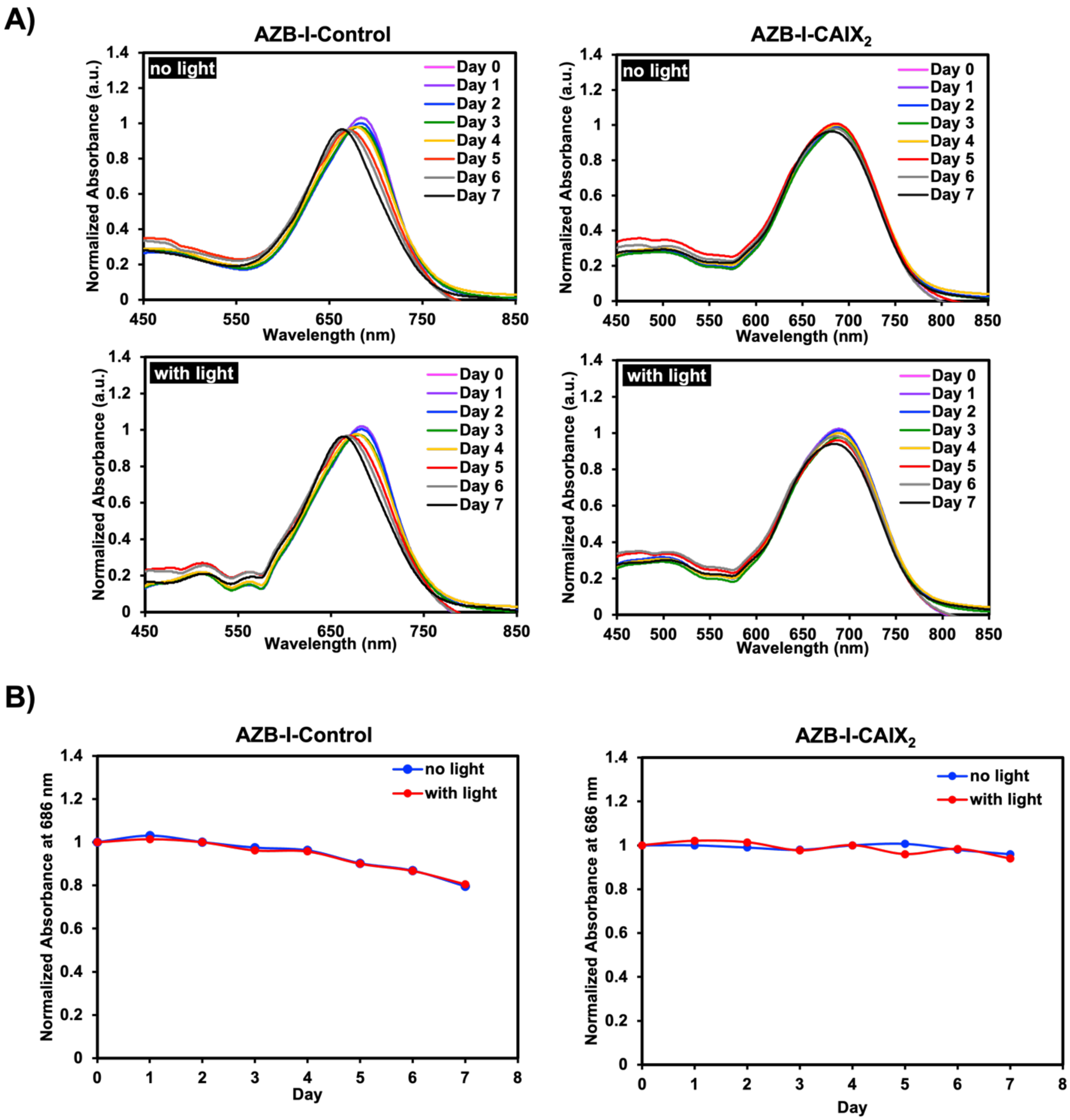

Fig S5. Stability of **AZB-I-CAIX$_2$** and **AZB-I-Control** in fetal bovine serum (FBS) under light and dark conditions. A) Normalized absorbance measuring for 7 days. B) Normalized intensities at 686 nm measuring for 7 days.

7. Biological studies experiments

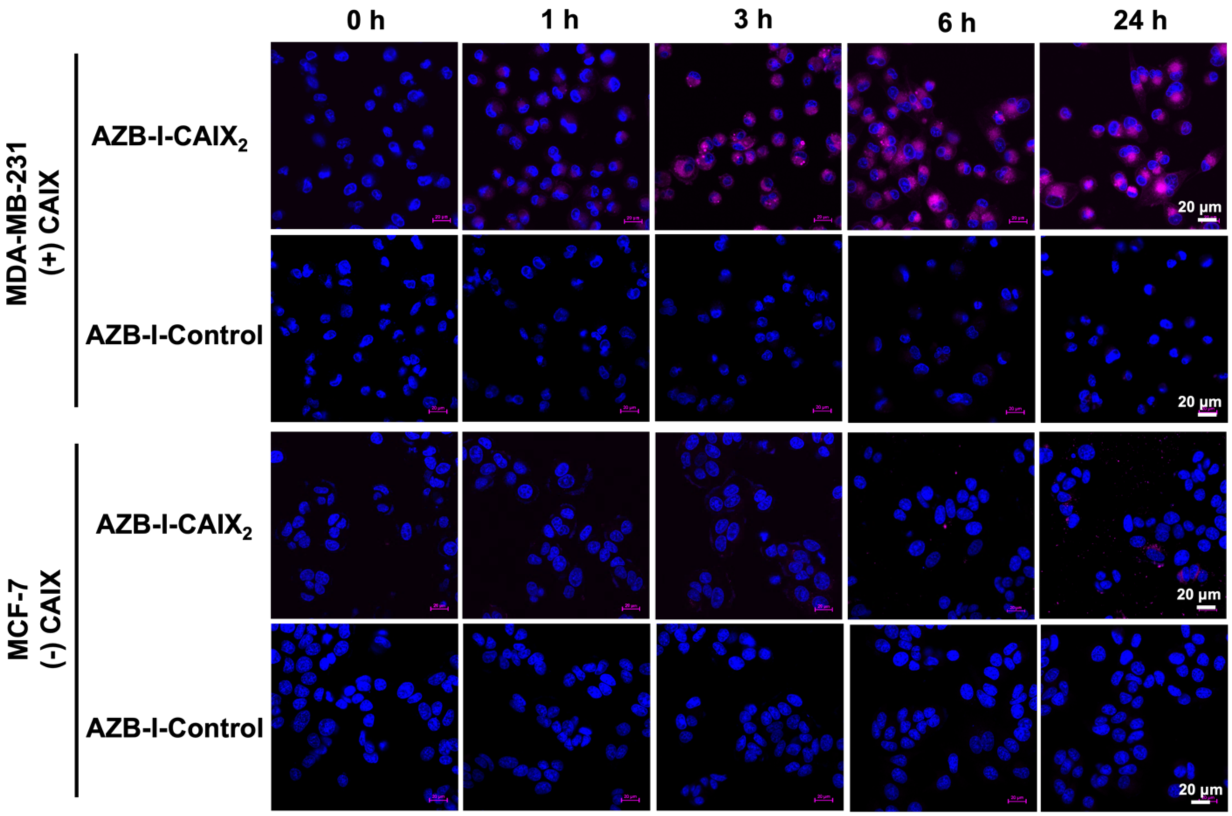

Fig S6. Confocal image of CAIX positive (MDA-MB-231) and negative (MCF-7) cell lines by treating 5 μM of **AZB-I-CAIX$_2$** and **AZB-I-Control** for 0, 1, 3, 6, and 24 h.

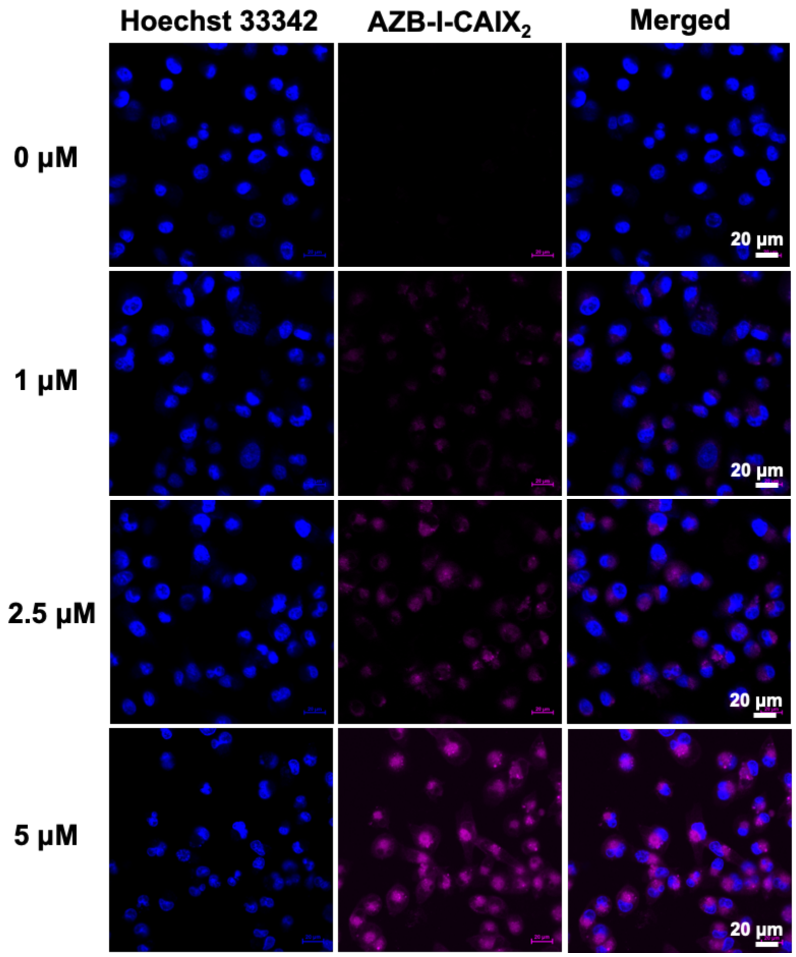

Fig S7. Confocal image of dose-dependent cell internalization in MDA-MB-231 cells by treating varying concentrations of **AZB-I-CAIX$_2$** (0, 1, 2.5, and 5 μM) for 6 h.

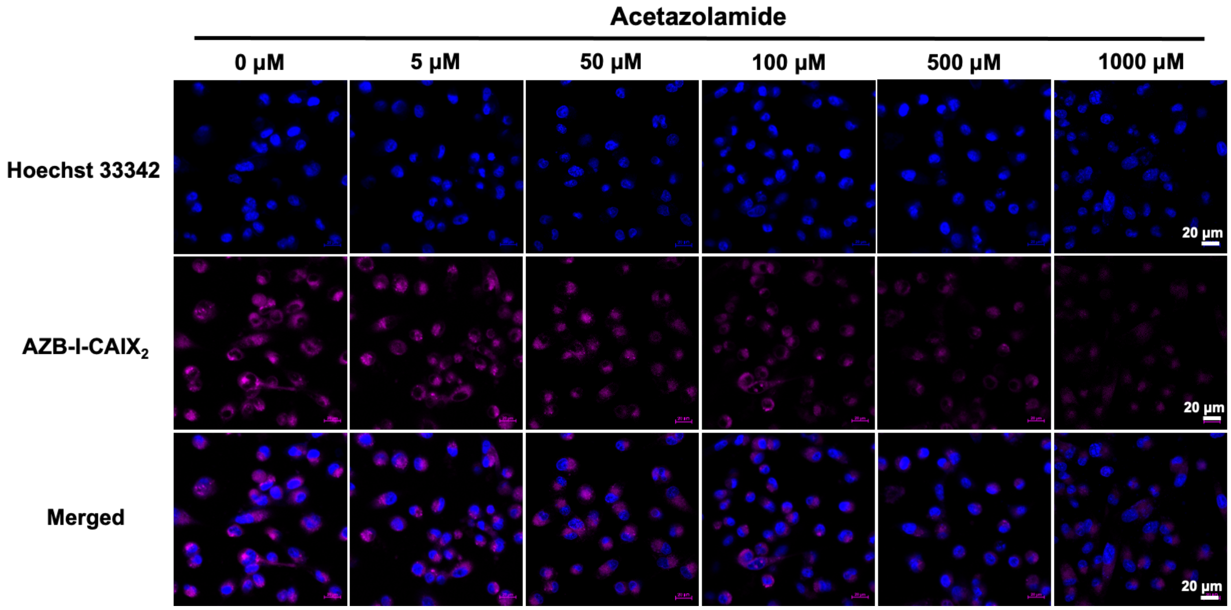

Fig S8. Confocal image of CAIX positive (MDA-MB-231) lines in CAIX inhibitor dose-dependent on competition effect by treating 5 μM of **AZB-I-CAIX$_2$** and 0, 5, 50, 100, 500, and 1000 μM of acetazolamide for 6 h.

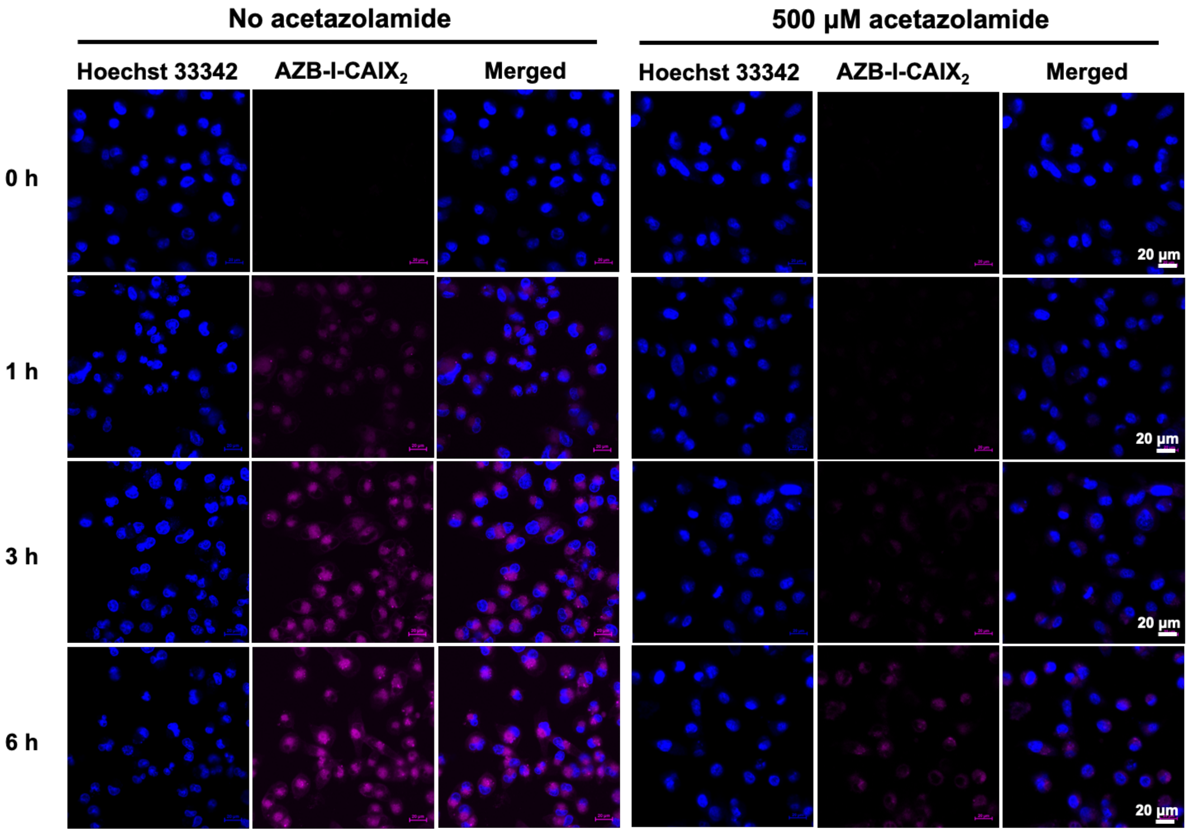

Fig S9. Confocal image of CAIX positive (MDA-MB-231) lines in time-dependent competition effect with CAIX inhibitor by treating 5 μM of **AZB-I-CAIX$_2$** and 500 μM of acetazolamide for 0, 1, 3, and 6 h.

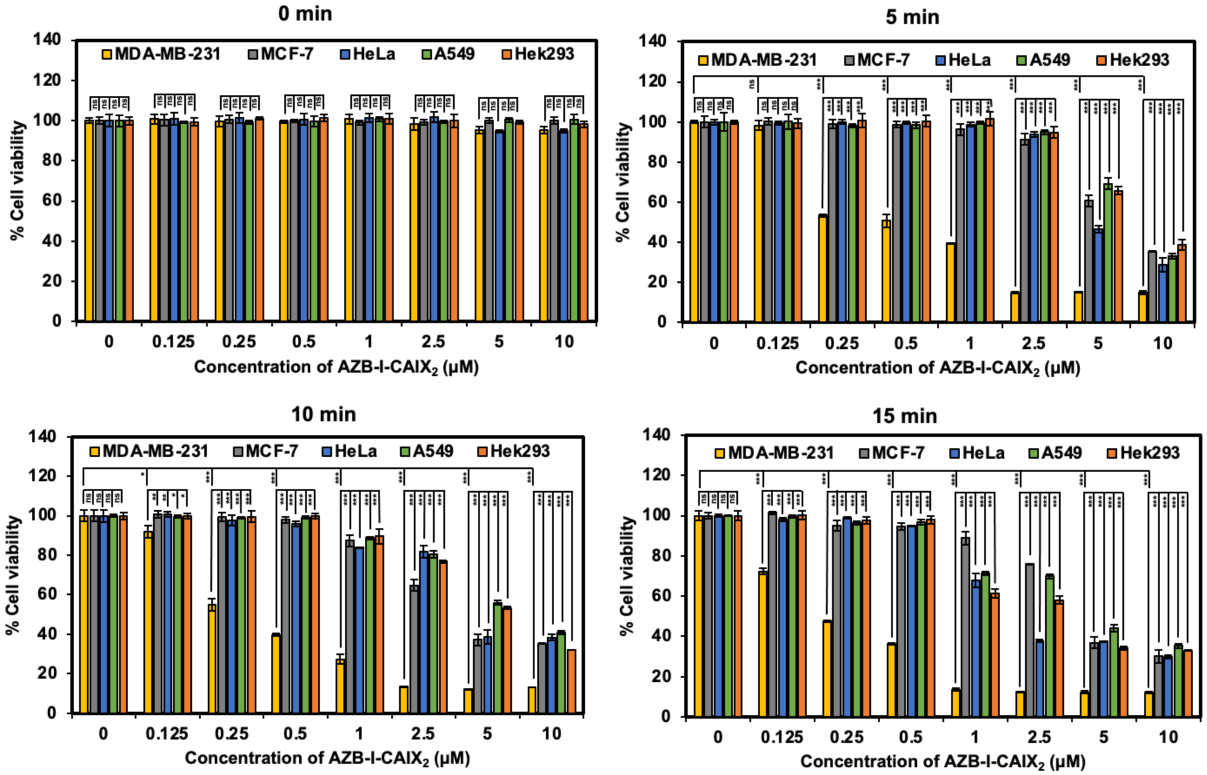

Fig S10. Relative cell viability of CAIX+ (MDA-MB-231) and CAIX- (MCF-7, HeLa, A549, HEK-293) cells exposed to **AZB-I-CAIX$_2$** with different light irradiation times (0, 5, 10, 15 min). The cells were incubated with various concentrations of **AZB-I-CAIX$_2$** (0–10 μM) for 6 h and irradiated with a lamp (660 nm, power density of 8.7 mW cm^{-2}). Statistical analysis: One-way ANOVA followed by Tukey's analysis was used for comparison between multiple groups using GrapPad Prism9 software. P values of less than 0.05 (95% confidence interval) are considered significant (ns $p < 0.12$, * $p < 0.033$, ** $p < 0.002$, *** $p < 0.001$).

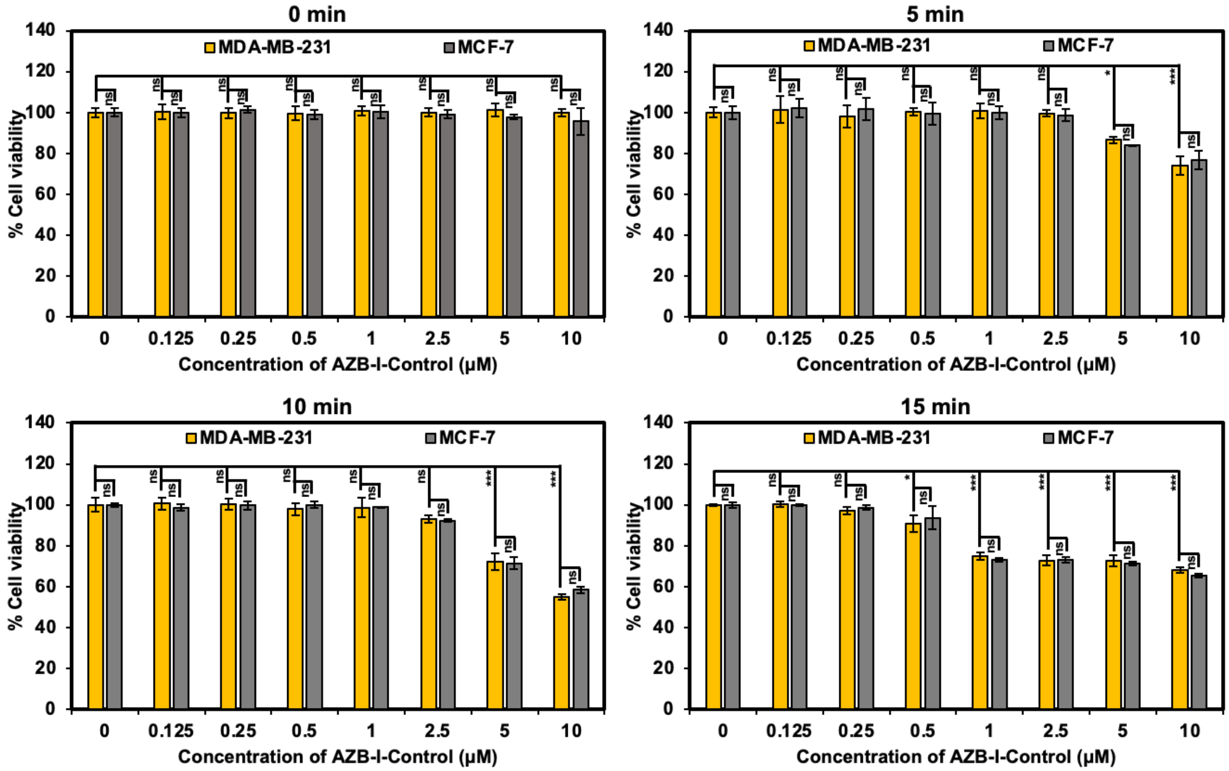

Fig S11. CAIX+ (MDA-MB-231) and CAIX- (MCF-7) cell lines were incubated with various concentrations of **AZB-I-Control** for 6 h and irradiated with a lamp (660 nm, power density of 8.7 mW cm^{-2}). Statistical analysis: One-way ANOVA followed by Tukey's analysis was used for comparison between multiple groups using GrapPad Prism9 software. P values of less than 0.05 (95% confidence interval) are considered significant (ns $p < 0.12$, * $p < 0.033$, ** $p < 0.002$, *** $p < 0.001$).

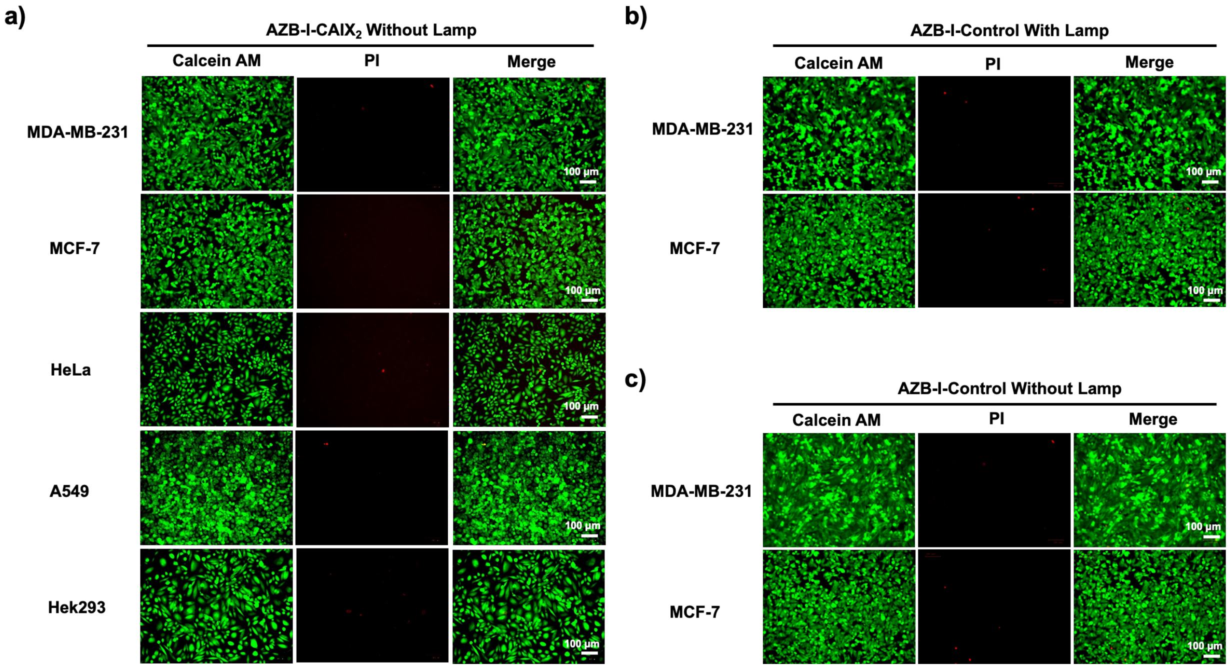

Fig S12. LIVE/DEAD staining of the cells. a) **AZB-I-CAIX$_2$** without lamp, b) **AZB-Control-I** with the lamp for 5 min, c) **AZB-Control-I** without lamp.

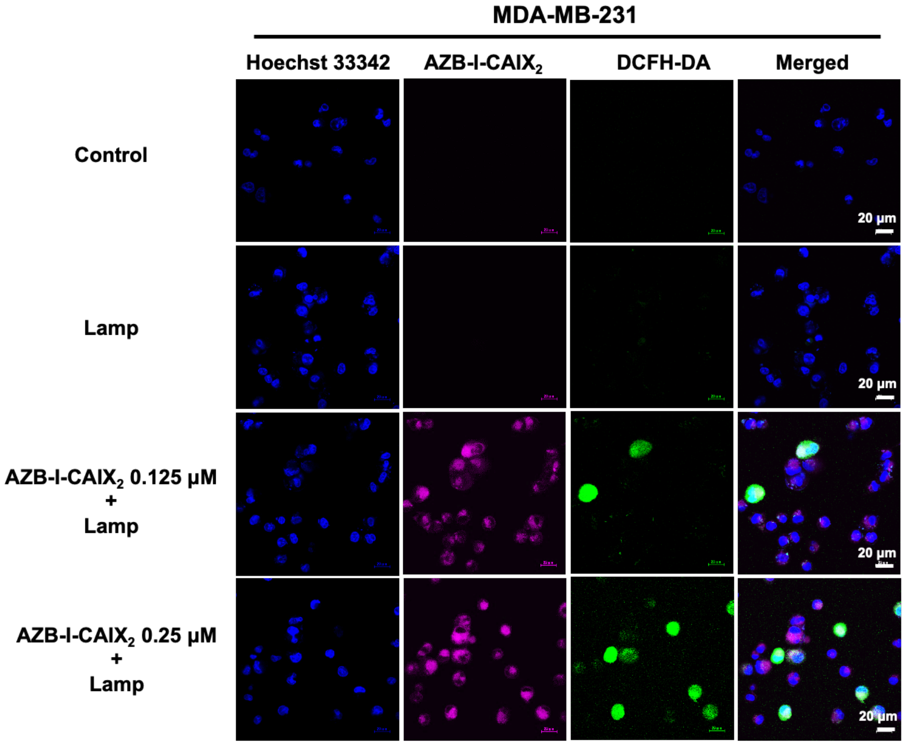

Fig S13. Confocal images of MDA-MB-231 cells incubated with **AZB-I-CAIX$_2$** (0.125 and 0.5 μM) and light irradiation for 10 min in the presence of ROS detection probe, DCFH-DA. The green emission signal indicated the existence of ROS inside the cells.

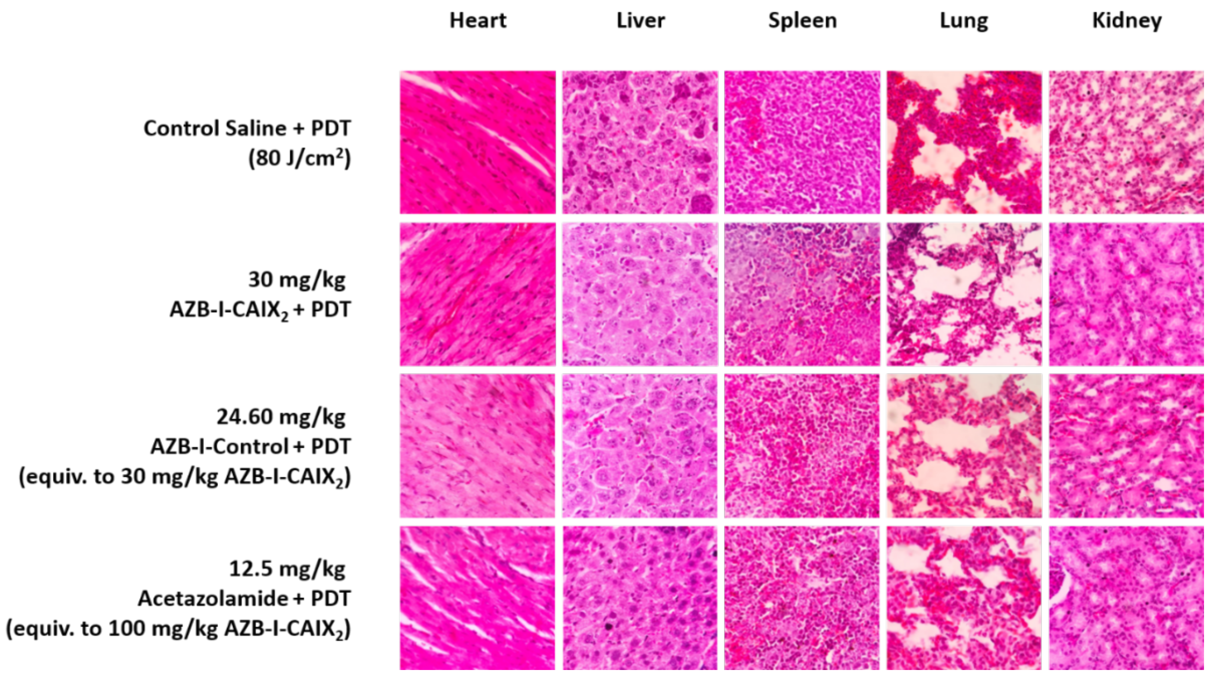

Fig S14. Histopathological H&E analysis of the major organs in different groups post-acute intravenous administration. Organs such as heart, liver, spleen, lung, and kidney were harvested from mice at 14 days post intravenous administration. The picture shown is the representative from each group with similar patterns. Magnification: x40.
